# The Interaction Mechanism of Intrinsically Disordered PP2A Inhibitor Proteins ARPP-16 and ARPP-19 With PP2A

**DOI:** 10.3389/fmolb.2021.650881

**Published:** 2021-03-26

**Authors:** Chandan Thapa, Pekka Roivas, Tatu Haataja, Perttu Permi, Ulla Pentikäinen

**Affiliations:** ^1^Department of Biological and Environmental Science and Nanoscience Center, University of Jyvaskyla, Jyvaskyla, Finland; ^2^Institute of Biomedicine, University of Turku, Turku, Finland; ^3^Turku BioScience Centre, University of Turku, Turku, Finland; ^4^Department of Chemistry and Nanoscience Center, University of Jyvaskyla, Jyvaskyla, Finland

**Keywords:** intrinsically disordered proteins, NMR spectroscopy, SAXS, PP2A, protein-protein interaction, PP2A inhibitor proteins, ARPP-19, ARPP-16

## Abstract

Protein phosphatase 2A (PP2A) activity is critical for maintaining normal physiological cellular functions. PP2A is inhibited by endogenous inhibitor proteins in several pathological conditions including cancer. A PP2A inhibitor protein, ARPP-19, has recently been connected to several human cancer types. Accordingly, the knowledge about ARPP-19—PP2A inhibition mechanism is crucial for the understanding the disease development and the therapeutic targeting of ARPP-19—PP2A. Here, we show the first structural characterization of ARPP-19, and its splice variant ARPP-16 using NMR spectroscopy, and SAXS. The results reveal that both ARPP proteins are intrinsically disordered but contain transient secondary structure elements. The interaction mechanism of ARPP-16/19 with PP2A was investigated using microscale thermophoresis and NMR spectroscopy. Our results suggest that ARPP—PP2A A-subunit interaction is mediated by linear motif and has modest affinity whereas, the interaction of ARPPs with B56-subunit is weak and transient. Like many IDPs, ARPPs are promiscuous binders that transiently interact with PP2A A- and B56 subunits using multiple interaction motifs. In summary, our results provide a good starting point for future studies and development of therapeutics that block ARPP-PP2A interactions.

## Introduction

Protein phosphorylation is a dynamic process regulating the functionalities of proteins involved in many cell signaling processes. The equilibrium of protein phosphorylation is achieved through opposing activities of protein kinases and phosphatases. Perturbations in the activities of both enzyme classes promote the development of many human diseases (Sangodkar et al., 2017). Protein phosphatase 2A (PP2A) is one of the major cellular Serine/Threonine phosphatases. Its proper activity is critical for maintaining normal physiological cellular functions. PP2A inhibition has been observed to contribute to pathogenesis in many diseases such as cancer, cardiovascular disease, diabetes, neurodegenerative disease (e.g., Alzheimer’s and Parkinson’s disease), and developmental conditions involving intellectual disability (Mazhar et al., 2019). Therefore, the understanding of the PP2A inhibition mechanism and the therapeutic targeting of PP2A has become a tempting area of research with promising potential for clinical impact.

In cancer, the PP2A inactivation removes its tumor suppressor activity, which inhibits the activity of several critical oncogenic signaling pathways preventing the transformation of normal human cells into cancerous cells (Kauko and Westermarck, 2018). The most prevalent mode for the PP2A tumor suppressor activity inhibition in cancer is the overexpression of PP2A inhibitor proteins (Meeusen and Janssens, 2018). In recent years, several otherwise unrelated cellular proteins that inhibit PP2A activity in tumors, such as Cancerous Inhibitor of Protein Phosphatase 2A (CIP2A) (Junttila et al., 2007), SET (SET nuclear oncogene) (Li et al., 1996), T-cell Immunomodulator Protein (TIP) (Li et al., 1996), Phosphatase Methyl Esterase-1 (PME-1) (Ogris et al., 1999), and cAMP-regulated phosphoprotein 19 (ARPP-19) (Gharbi-Ayachi et al., 2010; Mochida et al., 2010; Song et al., 2014; Jiang et al., 2016) have been identified. The essential role of PP2A inhibition in human cell transformation and cancer progression makes the identification of PP2A-inhibitory mechanism vital for the understanding the molecular bases of cancer (Kauko and Westermarck, 2018; O’Connor et al., 2018). So far, it is clear that different PP2A inhibitor proteins modulate PP2A activity by different, highly sophisticated mechanisms. A better understanding of these mechanisms is, however, needed to understand the physiological and pathological conditions. Additional insight into the structural, molecular, and biological framework is also required to provide a foundation for the development of novel and clinically feasible PP2A targeted therapies (O’Connor et al., 2018).

**FIGURE 1:**
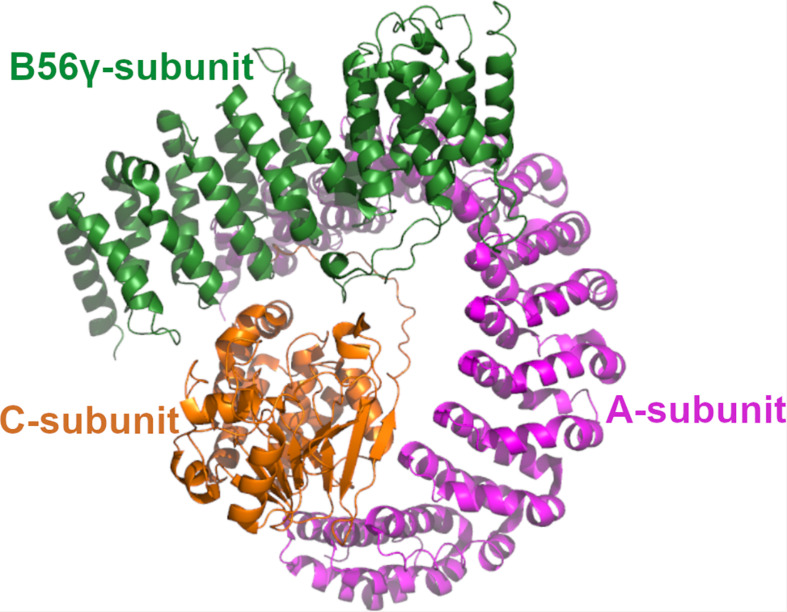
The structure of PP2A. The 3D structure of PP2A. The scaffolding A-subunit (PR65α) is shown with magenta, regulatory B56γ subunit shown with green and the catalytic Cα-subunit with orange. The coordinates are taken from 2npp.pdb.

Protein phosphatase 2A is a heterotrimeric enzyme complex consisting of the scaffolding A-subunit, the catalytic C-subunit and the regulatory B-subunit (Figure 1; Sangodkar et al., 2017; Kauko and Westermarck, 2018). The A- and C-subunit form the core enzyme which interacts with the B-subunit to create the holoenzyme. Both A- and C-subunits have two homologous isoforms, α and β. The B-subunits are a structurally more diverse group, consisting of four structurally distinct families, B55, PR72, B56, and the Striatin family, that influence the PP2A substrate specificity (Westermarck and Hahn, 2008). In addition, each B-subunit family has multiple isoforms encoded by different genes. Accordingly, the different combinations of the A-, B-, and C-subunits give rise to a large number of distinct trimers. Although different B-subunits have partly redundant functions, different PP2A trimer combination may have specific physiological role due to cell type, tissue specific expression of subunits, and selective interaction between different B-subunits and their target proteins. Some B-subunit containing PP2A enzymes have been connected to tumor suppression as they function as antagonist in oncogenic signaling pathways. Well known tumor suppressor B-subunits and their identified targets are B56α-mediated regulation of MYC, and B55α and B56γ mediated negative regulation of AKT kinase phosphorylation. Also B56ε has been reported to suppresses p53-independent apoptosis but induces p53-dependent apoptosis (Jin et al., 2010) and a high frequency of PP2A B56ε downregulation is observed in AML cell lines (Cristóbal et al., 2013) that could be associated, at least in part, with p53 deregulation.

ARPP-19 is a crucial regulator of the cell division. The protein kinase Greatwall (Gwl) phosphorylated ARPP-19 inhibits PP2A prompting a correct timing and progression of mitosis (Gharbi-Ayachi et al., 2010; Mochida et al., 2010). Also, cyclin B-Cdc2 has been reported directly to phosphorylate ARPP-19 to inhibit PP2A in a mitotic cycle (Okumura et al., 2014). During recent years, the importance of ARPP-19 as a key cancerous PP2A inhibitor protein has emerged (Song et al., 2014; Jiang et al., 2016; Mäkelä et al., 2019). Increased expression of ARRP-19 has been reported in several cancer types such as hepatocellular carcinoma (HCC) (Song et al., 2014), human glioma (Jiang et al., 2016), and acute myeloid leukemia (AML) (Mäkelä et al., 2019). Glioma is one of the most common and aggressive types of human brain tumor. Currently, the conventional treatment for glioma is surgical resection and the adjuvant chemotherapy with or without radiotherapy. Despite advances in surgery and adjuvant therapy, the 5-year survival rate is lower than 5% (Jiang et al., 2016). HCC is, in turn, the most common of primary liver cancer in adults. Patients diagnosed with HCC usually have a poor prognosis because of its aggressive nature. Currently, no effective treatment is available for HCC patients at advanced stage (Jiang et al., 2016). As for AML, it is the most common acute leukemia affecting adults, progressing rapidly and aggressively. In 2010, 85% of the patients with AML obtained complete remission, but only 40% can be fully cured (Petersdorf et al., 2013; Döhner et al., 2015). Altogether, all ARPP-19 related cancer types are very aggressive with poor outcome, and new treatments are urgently needed. To this end, the structural and molecular level characterization of the ARPP-19 mediated PP2A inhibition mechanism is required.

The well-defined three-dimensional (3D) structure is still often thought to prerequisite for protein function. However, intrinsically disordered proteins (IDPs), which form a substantial part of protein kingdom, have been proven to have unique structure-independent functions. IDPs are characterized by the biased amino acid composition with low complexity and high structural flexibility. Although IDPs do not form a single stable 3D structure, they can form transient conformational ensembles. IDPs are involved in protein-protein interactions having a central role in the regulation of signaling pathways and key cellular processes. Upon binding to their targets, many IDPs undergo a disorder-to-order transition to form well-defined structures while bound with their targets. IDPs tend to follow “templated folding” mechanism where the structure of the transition state of the folding is dictated by the binding partner. Many IDPs also contain multiple interaction motifs to be able to bind with multiple proteins at the same time to form larger complexes (Berlow et al., 2015; Toto et al., 2020). Accordingly, it is not surprising that IDPs are linked to many pathogenesis of human diseases such as cancer, diabetes, neurodegeneration, cardiovascular disease, and amyloids (Uversky, 2020).

Here we have characterized structural properties of PP2A inhibitory protein ARPP-19, and its splice variant ARPP-16 by combining nuclear magnetic resonance (NMR) spectroscopy and small-angle X-ray scattering (SAXS). Our results reveal that both ARPP-16 and ARPP-19 are IDPs, i.e., they do not form stable 3D structures but exhibit an ensemble of transient conformations, of which the compact ones are more favored than extended ones. We have also characterized so far unknown interaction mechanism of ARPP-16/19 with PP2A, including the affinity of different tumor suppressor B56 subunits and A-subunit to both ARPP proteins, as well as identified the A-subunit binding site at ARPP-16 and ARPP-19. Altogether, our results provide (1) the first molecular-level information about ARPP mediated PP2A inhibition, and (2) new knowledge about the interactions between ARPP proteins and the PP2A regulatory B-subunits.

## Materials and Methods

### *In silico* Analysis of ARPP-16/19

IUPred2A is a unified platform that predicts the disordered protein regions and disordered binding regions using IUPred2 and ANCHOR2 tools, respectively. The UniProt identifier codes of ARPP-19 and ARPP-16 served as input and predictions were performed using default setting. IUPred2 calculates pairwise energy profile along the sequence and are transformed into a probabilistic score between 0 and 1. An IUPred score of 0 and 1 reflects complete order and disorder state, respectively, and residue with the score above 0.5 are regarded as disordered (Mészáros et al., 2018; Erdős and Dosztányi, 2020). ANCHOR2 also uses an energy estimation approach similar to IUPred to predict the degree of disorder along with two additional energy estimation terms. The additional energy estimation terms calculate energy associated with interaction with a globular protein partner assuming that disordered binding regions cannot form favorable interchain interaction on their own to induce folding but can acquire stabilizing energy after interacting with a globular protein partner. Similar to IUPred2, energy estimates are transformed into a probability score between zero and one, indicating the likelihood of residue to be a part of the disordered binding region and residues with the score above 0.5 are considered to be in the disordered binding regions (Mészáros et al., 2018; Erdős and Dosztányi, 2020).

### The Expression and Purification of the Recombinant Proteins

#### ARPP-16 and ARPP-19

The sequence of the synthetic gene of human ARPP-19 (UniProt accession number: P56211-1) and its splice variant ARPP-16 (P56211-2), were designed according to *E. coli* codon usage. ARPP-19 was PCR amplified and cloned to a modified pGEX vector (GE Healthcare, Chicago, IL, United States), whereas PCR amplified ARPP-16 was cloned to a pGTvL1-SGC vector (Structural Genomics Consortium, University of Oxford, Oxford, United Kingdom) according to the ligation-independent cloning method (Savitsky et al., 2010). The phosphomimicking mutants of ARPP-16 (S46E and S88E) and ARPP-19 (S62E and S104E) were generated using QuikChange II Site-Directed Mutagenesis Kit (Agilent Technologies, Santa Clara, CA, United States). All expression plasmids of ARPP-19 and ARPP-16 were verified by sequencing. Glutathione *S*-transferase (GST) fusion proteins were produced in Terrific Broth (2.4% w/v yeast extract, 1.2% w/v tryptone, 0.5% w/v glycerol, 0.017 M KH_2_PO_4_, 0.072 M K_2_HPO_4_) by the addition of isopropyl-β-D-1-thiogalactopyranoside to 0.4 mM at 18°C for 20 h using *E. Coli* BL21 Gold cells. The cells were lysed using EmulsiFlex-C3 homogenizer (Avestin, Ottawa, ON, Canada) and subsequently centrifuged at 35,000 × *g* for 30 min at 4°C to clear the lysate. The GST fusion proteins were purified with Protino Glutathione Agarose 4B (Macherey-Nagel, Düren, Germany) and GST was cleaved by Tobacco Etch Virus (TEV) protease (Invitrogen, Life Technologies, Carlsbad, CA, United States) at 4 °C for 16 h. The TEV protease cleavage extended ARPP-19 constructs in the N-terminal by four amino acid residues, G, A, M, and G, and ARPP-16 constructs by one amino acid residue, S. The proteins were further purified by size exclusion chromatography (SEC) with a HiLoad 26/60 Superdex 200 pg column (GE Healthcare, Chicago, IL, United States) in SEC buffer (50 mM NaH_2_PO_4_, pH 6.8, 100 mM KCl, and 1 mM DTT) using an ÄKTA pure chromatography system (GE Healthcare). The proteins were concentrated with Amicon ultracentrifugal 3K filter device (MilliporeSigma, Burlington, MA, United States). The homodispersity of the proteins was verified with SEC-MALS and sodium dodecyl sulphate polyacrylamide gel electrophoresis (SDS-PAGE).

The ^15^N and ^13^C-labeled ARPP-16/19 constructs were expressed in *E. coli* BL21 Gold cells in standard M9 minimal medium using 1 g/l ^15^N NH_4_Cl and 2 g/l ^13^C D-glucose (Cambridge Isotope Laboratories, Tewksbury, MA, United States) as a sole nitrogen and carbon sources, respectively. The proteins were purified in 50 mM NaH_2_PO_4_, pH 6.5, 100 mM NaCl, 1 mM DTT using the same protocol as described above for the unlabeled proteins. The protein samples were supplemented with 5% D_2_O prior to the measurements.

#### The PP2A A-Subunit

The PP2A scaffolding subunit PR65α (UniProt accession number: AK7B7), both the full length and the N-terminal fragment of PR65α (Heat repeats 1–7, corresponding amino acids 1–274) were cloned to the pGTvL1-SGC vector (Structural Genomics Consortium, University of Oxford, Oxford, United Kingdom) according to the ligation-independent cloning method (Savitsky et al., 2010). The expression plasmids were verified by sequencing. The recombinant proteins were expressed and purified in the same way as the unlabeled ARPP proteins. The proteins were concentrated with Amicon ultracentrifugal 30K filter device (MilliporeSigma, Burlington, MA, United States).

#### The PP2A Regulatory Subunits

The regulatory subunits of PP2A; B56α (UniProt accession number: Q15172-1), B56δ (Q14738-1), B56γ (Q13362-1), and B56ε (Q16537-1) were cloned to the pGTvL1-SGC vector (Structural Genomics Consortium, University of Oxford, Oxford, United Kingdom) according to the ligation-independent cloning method (Savitsky et al., 2010). The expression plasmids of the above mentioned regulatory subunits were verified by sequencing. The recombinant proteins were expressed in the same way as the unlabeled ARPP proteins. The cells were lysed by sonication on ice (Sonoplus 4000) at 40% amplitude (4x, 1 s pulse on and 1 s pulse off) for 1 min. The GST fusion proteins were purified similarly as the ARPP proteins using the following SEC buffers: 50 mM NaH_2_PO_4_, 75 mM KCl, 0.03% CHAPS, 1 mM DTT, pH 6.8 (B56δ), and pH 7.5 (B56α, B56γ, and B56ε). The TEV protease cleavage extended the constructs in the N-terminal by one amino acid residue, S. The proteins were concentrated with Amicon ultracentrifugal 30K filter device (MilliporeSigma, Burlington, MA, United States).

### SAXS Analysis of ARPP-16/19

The SAXS data were collected at the ESRF (European Synchrotron Radiation Facility) BM29 (Pernot et al., 2013) beamline in Grenoble, France. A Pilatus 1 M image plate was used, sample detector distance 2.85 m and wavelength 0.10 Å, covering the momentum transfer 0.01 < *q* > 5 nm^–1^, where *q* = 4π sin (θ)/λ, and 2θ is scattering angle. Protein concentrations used in the data acquisition were 1, 3, 5, 7, 10, and 12.5 mg/ml. The proteins were diluted in the SEC buffer supplemented with 10 mM DTT. The data were processed using the standard procedures of the ATSAS program package (European Molecular Biology Laboratory, Hamburg, Germany) (Franke et al., 2017). The radius of gyration (*R*_*g*_) and a maximum dimension of the particle (*D*_*max*_) were estimated from Guinier analysis performed in PRIMUS (Konarev et al., 2003) and distance distribution function was calculated using DATGNOM (Petoukhov et al., 2007). The Kratky plot [*I*_(_*_*s*_*_)_^∗^*s*^2^ vs. *s*] (Durand et al., 2010) was used to evaluate the flexibility of the proteins. The flexibility of the ARPP proteins was studied using ensemble optimized method (EOM) version 2.0 (Bernadó et al., 2007; Tria et al., 2015) on ATSAS online. In the EOM calculation, the data from single protein concentration (7 mg/ml) was used.

### The Determination of the Binding Affinity Between the PP2A Subunits and ARPP-16/19

The PP2A subunits PR65α, B56α, B56δ, B56γ, and B56ε were labeled using Monolith NT Protein labeling kit red NHS, NT-647-NHS fluorescent dye (Cat no. L001, NanoTemper Technologies) and applied at the final concentration of 20 nM in SEC buffer having 0.05% Tween-20. A 12-point twofold dilution series of unlabeled proteins, ARPP-19, ARPP-16 and their phosphomutants were mixed with labeled proteins. The final concentrations of the ARPP proteins range from 3 to 50 nM. Microscale thermophoresis (MST) experiments were conducted in triplicate using a Monolith NT Automated system (NanoTemper Technologies GmbH, Munich) to determine the binding affinity between ARPP-16/19 and the PP2A subunits. The dissociation constant was then calculated using a single-site binding model to fit the curve and errors with standard error of mean (SEM) using GraphPad Prism version 7.0 for Windows (GraphPad Software Inc., La Jolla, CA, United States).

### NMR Data Collection and Processing

All spectra were obtained at 25°C using a Bruker Avance III HD 800 MHz NMR spectrometer equipped with a 5-mm triple resonance inverse TCI CryoProbe (TCI ^1^H-^13^C/^15^N-^2^H + Z gradient). The double- and triple resonance experiments performed for the sequence-specific backbone and partial side-chain assignments included two-dimensional (2D) ^15^N-HSQC (Kay et al., 1992), ^13^C-CON (Bermel et al., 2005), and 3D CBCA(CO)NH, HNCACB (Yamazaki et al., 1994), HNCO (Kay et al., 1994), iHNCO (Mäntylahti et al., 2009), iHA(CA)NCO, HA(CA)CON (Mäntylahti et al., 2010), and (HACA)CON(CA)HA (Mäntylahti et al., 2011) spectra. Spin-lattice relaxation rates (^15^N *R*_1_), spin-spin relaxation rates (^15^N *R*_2_) and steady-state heteronuclear {^1^H}-^15^N NOEs were determined using the method described in Farrow et al. (1994). For the ^15^N *R*_1_ and *R_2_*, ten 2D ^15^N-HSQC spectra, with the relaxation delays of 20, 100, 200, 300, 400, 600, 800, 1,000, 1,200, and 1,400 ms, and 16.96, 50.88, 84.8, 118.72, 135.68, 152.64, 169.6, 203.52, 237.44, and 271.36 ms were acquired, respectively. For {^1^H}-^15^N NOE values, NOE mixing time of 10 s was used. The spectra were processed using TopSpin 3.5 software package (Bruker Corporation, Billerica, MA, United States) and analyzed using NMRFAM-Sparky 3.13 (Lee et al., 2014).

The chemical shifts of ^13^Cα, ^13^Cβ, and ^1^Hα were analyzed with the Secondary Structure Propensity (SSP) software (Marsh et al., 2006) to calculate SSP scores for each ARPP-19/ARPP-16 residue, using the default settings. ENSEMBLE software suite (Krzeminski et al., 2013) was used to determine and analyze the weighted ensemble of structures using the chemical shifts (Cα, Cβ and, HN shifts), the ^15^N R_2_ relaxation, and the SAXS data. The chemical shifts of ARPP-16 and ARPP-19 have been deposited to Biological Magnetic Resonance Data Bank under the accession number 27911 and 27912, respectively (Thapa et al., 2020).

### The Determination of the PP2A A-Subunit Binding Site in ARPP-16/19 by NMR

The ^15^N-labeled ARPP proteins were titrated with the unlabeled A-subunit of PP2A. The proportion of ARPP-16 and the A-subunit of PP2A (ARPP-16: PP2A A-subunit) used in the titration were 1:0.5 (200:100 μM), 1:1 (162:170 μM), 1:2 (200:400 μM) and 1:3 (160:480 μM). The proportion of ARPP-19 and the A-subunit of PP2A (ARPP-19: PP2A A-subunit) used in the titration tests were 1:0.5 (200:100 μM), 1:1 (180:180 μM), 1:2 (160:320 μM) and 1:3 (140:450 μM). All proteins were purified in 50 mM NaH_2_PO_4_, 100 mM NaCl, 1 mM DTT, pH 6.8. ^15^N-HSQC titration experiments were performed on a Bruker Avance III HD 800 MHz NMR spectrometer at 25°C. All spectra were processed with TopSpin 3.5 software package (Bruker Corporation, Billerica, MA, United States) and analyzed using NMRFAM-Sparky 3.13 (Lee et al., 2014).

## Results

### *In silico* Analysis of ARPP-16/19

**FIGURE 2:**
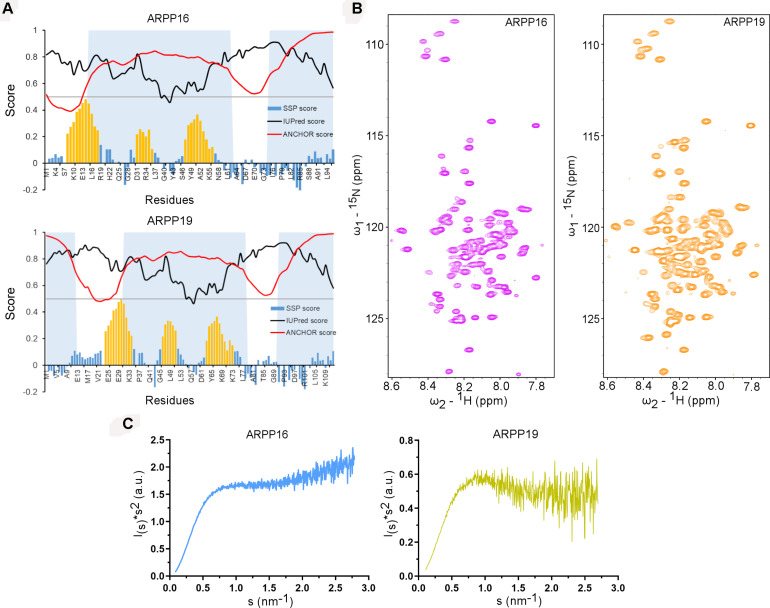
ARPP-19 and ARPP-16 are intrinsically disordered proteins having propensity to form three transient α-helices. **(A)** IUPRED2A analysis and secondary structure propensity (SSP) calculation of ARPPs. IUPred2 and Anchor2 score are represented with black and red curves, respectively. The disordered prediction score in black highlights disordered nature of ARPPs and disordered binding region prediction score in red suggests presence of multiple disordered binding regions which are shown as shaded region. SSP score for ARPPs calculated using ^1^Hα, ^13^Cα, and ^13^Cβ chemical shift shows that both ARPPs have propensity to form three α-helices. The positions of α-helices are highlighted with orange color. **(B)** The cross peaks in the 2D ^15^N-HSQC spectra of uniformly ^15^N labeled ARPPs are highly overlapped because of the degeneracy of the proton spectra dispersion indicating that the ARPPs lacks well-defined 3D structure. **(C)** The Kratky plot calculated from SAXS data exhibits rising curve with increasing angle, which corresponds to the scattering pattern of IDPs.

The main objective of the present work is the structural characterization of ARPP-16 and ARPP-19 and the determination of the regions in ARPP proteins that are involved in binding to PP2A. ARPP-16 and ARPP-19 have otherwise identical amino acid sequence but ARPP-19 has extra 16 amino acids in the N-terminus (Supplementary Figure 1). The sequence analysis shows the enrichment of hydrophilic and charged residues as well as prolines (Supplementary Figure 1) (Thapa et al., 2020), which are elementary attributes of intrinsically disordered proteins (Hazy and Tompa, 2009; Uversky, 2010). To get more information about the level of disorder, the number of potential binding regions, and their locations, we performed sequence analysis *in silico* using IUPred2A tool (Mészáros et al., 2018; Erdős and Dosztányi, 2020). According to IUPred2A analysis, ARPPs are predicted to be fully disordered (Figure 2A). The presence of multiple disordered binding regions was also predicted in both proteins; residues 1–12, 31–78, and 91–112 in ARPP-19, and residues 15–62 and 75–96 in ARPP-16, which corresponds the regions 31–78 and 91–112 in ARPP-19 (Supplementary Figure 1 and Figure 2A).

### ARPP-16/19 Are Intrinsically Disordered Proteins

To glean detailed molecular-level information on ARPP-16/19, we employed various biophysical tools, e.g., NMR spectroscopy and SAXS assays for structural characterization of ARPP-16 and ARPP-19. Initially, to probe residue-level information on the structural order of the ARPP proteins, we performed 2D ^1^H, ^15^N correlation experiment (^15^N HSQC; heteronuclear single quantum coherence) NMR experiments. ^15^N HSQC spectra of both ARPP proteins exhibit low dispersion of amide proton (^1^HN) chemical shifts, spanning from 7.8 to 8.5 ppm, indicating structural disorder (Figure 2B). The performed SAXS experiments gave further support to structural disorder. The SAXS profile obtained for ARPP-16 and ARPP-19 presents a smooth curves with essentially no feature, a typical scattering profile of the disordered protein (Supplementary Figures 2, 3 and Supplementary Tables 1, 2). This was further supported by the Kratky analyses of the SAXS data which presents a plateau at large *s* values (Figure 2C). This behavior is observed for the intrinsically disordered proteins. To analyze more detailed structural properties of the ARPP proteins, we carried out sequence-specific backbone assignments using a suite of 2D and 3D correlation spectra (Thapa et al., 2020). The transient structural elements were then identified by using secondary chemical shifts (CS) and comparing them to random coil CS values. The results revealed the presence of three transient α-helices (Figure 2A).

**FIGURE 3:**
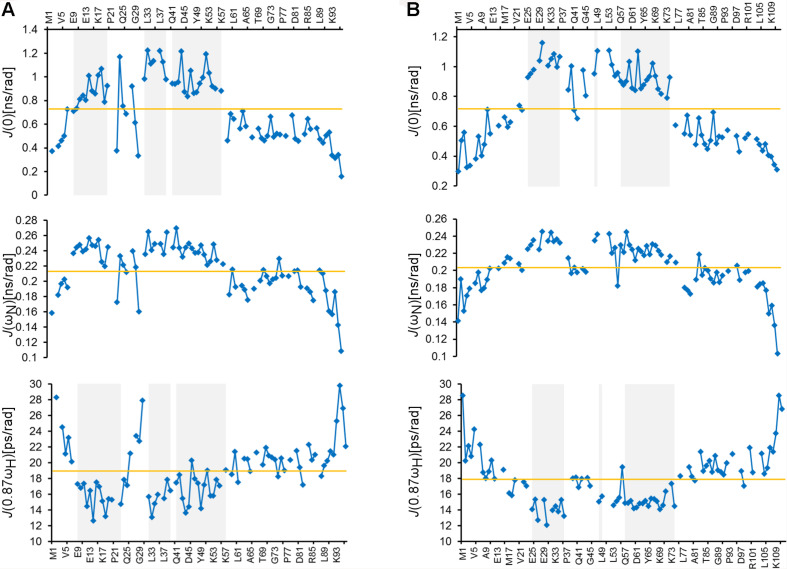
ARPPs have flexible N- and C-terminus whereas the core regions have restricted backbone motions. Plots for the spectral density function values at *J*(*0*), *J*(ω*_*N*_*), and *J*(*0.87*ω*_*H*_*) frequencies versus the residues of **(A)** ARPP-16 and **(B)** ARPP-19. Regions with restricted backbone motion are shaded.

To further study the structural properties of the ARPP proteins, we studied the ps-ns dynamics of the ARPP proteins using the T_1_ and T_2_^15^N spin relaxation and heteronuclear ^15^N NOE NMR experiments, which show that both N- and C-terminal amino acids are highly flexible. In contrast, the core regions of both ARPP proteins are more rigid (Supplementary Figure 4). The relaxation data were analyzed quantitatively using the spectral density mapping method (Farrow et al., 1994; Lefevre et al., 1996). The reduced spectral density mapping (RSDM) explains dynamics at three different frequencies, *J(0), J(*ω*_*N*_), and J(0.87*ω*_*H*_)*. For ARPP-16, the backbone motion of the regions with residues ^9^EKAEEAKLKARY^20^, ^32^FLRKRLQK^39^ and ^41^QKYFDSGDYNMAKAKMK^57^, are restricted as manifested by increased *J(0)* and decreased *J(0.87*ω*_*H*_)* values (Figure 3A). This is in good agreement with observed transient α-helices probed by the analysis of the secondary chemical shifts (Figure 2A). However, for the residues K42, S46, M51, and K57, the *J(0)* values increased, but the *J(0.87*ω*_*H*_)* values did not decrease significantly suggesting decreased backbone dynamics or conformational exchange at lower frequency. Similarly, the dynamics measured at the *J(0), J(*ω*_*N*_), and J(0.87*ω*_*H*_)* frequencies for ARPP-19 suggest that the regions with residues ^25^EKAEEAKLKARY^36^, ^48^FL^49^ and ^57^QKYFDSGDYNMAKAKMKNK^75^, have restricted backbone motion and correspond to the transient α-helical region of ARPP-19 (Figure 3B). The increased ps timescale dynamics, as demonstrated by the *J(0.87*ω*_*H*_)* values indicate the highly flexible nature of the N- and C-terminal regions of both ARPP-16 and ARPP-19.

### ARPP-16 and ARPP-19 Adopt Mainly Compact Conformation

**FIGURE 4:**
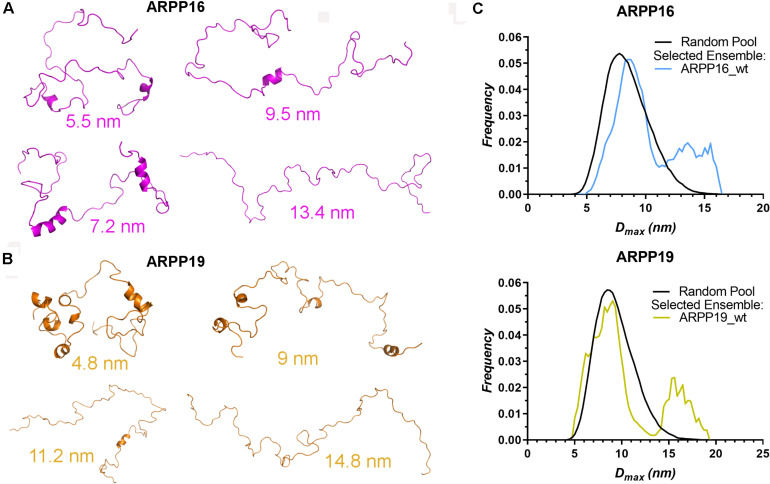
ARPPs obtain both compact and extended conformations. **(A**,**B)** The representation of different conformers of ARPP-16 and ARPP-19 calculated by ENSEMBLE program using chemical shifts (Cα, Cβ, and H), R_2_ relaxation rates from NMR spectroscopy and the SAXS data. The structure of different conformers obtained from ENSEMBLE calculation shows the conformational freedom of ARPPs. The D_max_ of each representative structure is shown. **(C)** Maximum distance (D_max_) distributions of ARPPs obtained from EOM analyses of the SAXS data plotted as function of frequency (a.u.) reveals that with both ARPPs, the compact conformations are more favored than extended ones.

The high flexibility of IDPs in comparison to proteins having a well-defined 3D structure, makes it difficult to interpret the structural properties using a single conformer approach. Therefore, the generation of the dynamic structural ensemble is essential to elucidate the structural properties of the IDPs. In this study, we employed the ENSEMBLE tool that utilizes an integrated approach of NMR and SAXS methods. The restraints used to calculate the ensemble of the structures that would best represent the conformational space of ARPP-16/19 are the NMR chemical shifts (Cα, Cβ, and HN), the ^15^N R_2_ relaxation rates, and the SAXS distance distribution data. The pool of the structures shows that ARPP-16/19 can adopt various conformations, of which some are more compact than others (Figures 4A,B). All conformers of the ARPP-16 and ARPP19 ensembles obtained from ENSEMBLE were clustered based on CαCα distance matrix RMSDs using the NMRClust algorithm (Kelley et al., 1996). The clustering shows that both ARPP proteins forms six ensembles composed of 8–20 models (Supplementary Figures 5, 6 and Supplementary Tables 3, 4). Although the individual models within each ensemble shows similarity based on CαCα distance matrix, the average CαCα RMSD of the ARPP-16 and ARPP-19 cluster is high, almost 20.0 Å, representing a large conformational difference among the models in the cluster. In other words, ARPP proteins have some preferred conformational ensembles but due to lack of the stable tertiary structure, the conformational flexibility is high. This is well-accordance with the SAXS data, as EOM calculations performed for the collected scattering data shows clearly, that both ARPP proteins adopt two different ensembles of conformations, compact and extended, of which the compact is the predominant (Figure 4C, Supplementary Figure 7, and Supplementary Table 5). Interestingly, the distribution of the conformations is not identical between ARPP-16 and ARPP-19. While with ARPP-19, there are two distinct sub-populations, the extended and compact one, with ARPP-16 the whole conformational space is more evenly covered (Figure 4C).

### The Phosphorylation of ARPP-16/19 Does Not Affect on Their Structural Properties

**FIGURE 5:**
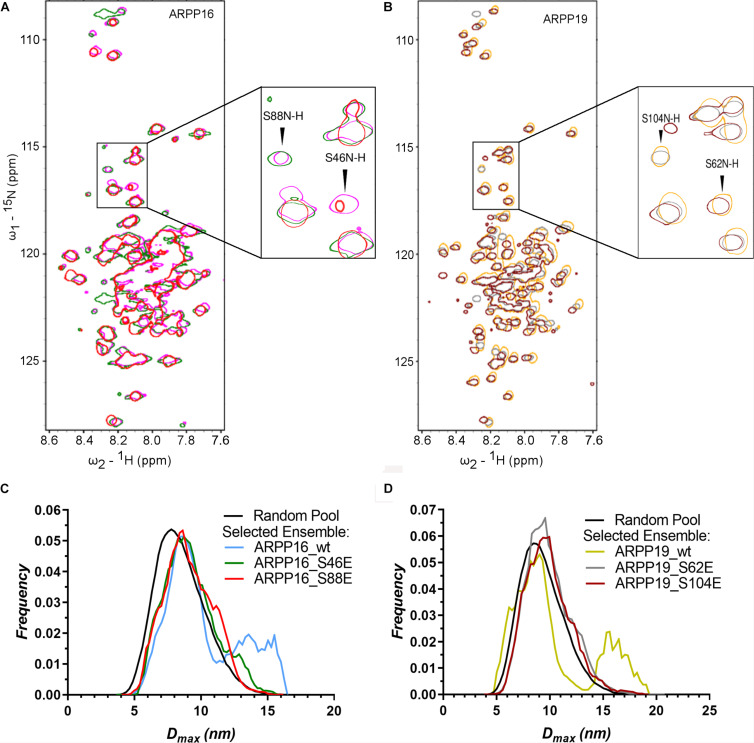
The phosphorylation does not have significant effect on the structural properties of ARPPs. **(A,B)** The overlay 2D ^15^N-HSQC spectra of uniformly ^15^N labeled ARPP WT and phosphomutants reveals that the phosphomimicking mutations have no significant effect on ARPPs’ structural properties. The cross peaks in the ^15^N-HSQC spectra of both ARPP-16 and ARPP-19 phosphomutants are highly similar to WT spectra, showing low dispersion of amide proton chemical shifts. This indicates that the ARPPs’ phosphomutants do not induce formation of well-defined 3D structure. **(A)** The spectrum of WT ARPP-16 is shown in magenta, ARPP-16 S46E and ARPP-16 S88E in green and red, respectively. **(B)** The spectrum of WT ARPP-19 is shown in orange, ARPP-19 S62E and ARPP-19 in gray and maroon, respectively. **(C,D)** D_max_ distributions of ARPPs’ WT and phosphomutants ARPPs obtained from EOM analyses of the SAXS data plotted as function of frequency (a.u.) reveals that with phosphomutants, the extended conformation is less favored than with WT.

**FIGURE 6:**
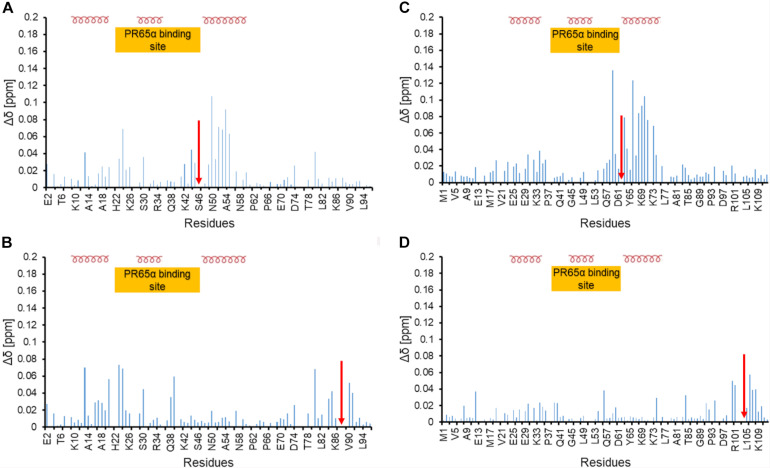
Chemical shift perturbation of phosphomimetic mutants of ARPP-16 and ARPP19 **(A)** and **(B)** Chemical shift perturbation due to S46E and S88E mutations, respectively. **(C,D)** Chemical shift perturbation due to S62E and S104E mutations, respectively. Δδ [ppm] refers to the combined HN and N chemical shift changes, according to the equation: Δδ(HN, N) = sqrt[(ΔδHN)^2^ + 0.2(ΔδN)^2^]. Red arrow indicates the phosphomimetic mutation site. Positions of α-helices and the A-subunit (PR65) binding site are shown.

Both ARPP proteins are phosphorylated at two different sites by specific kinases; ARPP-16 is phosphorylated by MAST3 kinases at Ser46 (Andrade et al., 2017) and PKA kinases at Ser88 (Dulubova et al., 2001). Similarly, ARPP-19 is phosphorylated by MAST3 and Gwl kinases at Ser62 (Mochida et al., 2010; Andrade et al., 2017) and PKA kinases at Ser104 (Dulubova et al., 2001). Also, cyclin B-Cdc2 has been reported to phosphorylate ARPP-19 (Okumura et al., 2014). MAST3 kinase phosphorylated ARPP-16 (Andrade et al., 2017) and Gwl phosphorylated ARPP-19 has been shown to inhibit PP2A function (Gharbi-Ayachi et al., 2010; Mochida et al., 2010; Andrade et al., 2017). In order to understand the effects of phosphorylation on the structural properties of ARPP-16/19, the phosphomimetic mutations S46E, S88E in ARPP-16, and S62E and S104E in ARPP-19 were generated, and the ^15^N-HSQC spectra were collected for all phosphomimicking mutants of both ARPP proteins. As can be seen from the overlaid ^15^N-HSQC spectra of phosphomimicking mutants and the WT ARPP proteins shown in Figure 5, the phosphomimicking mutation does not induce the formation of the stable tertiary structure or major conformational change but the ARPP phosphomimicking mutants remain disordered similar to WT ARPP proteins. However, as can be appreciated from Figure 6, the ARPP-16 S46E and ARPP-19 S62E mutations induce significant CSPs in the third transiently populated α-helix. The mutations in the C-terminal part i.e., ARPP-16 S88E and ARPP-19 S104E induce only minute CSPs in the polypeptide chain except for a few sequentially proximal residues. The EOM analyses performed for the SAXS data of the ARPP phosphomimicking mutants revealed that unlike ARPP WT, the ARPP phosphomimicks do not adopt two distinct conformational sub-populations, compact and extended one, yet the phosphomimicks have large average size due to the presence of extended conformations (Figures 5C,D, Supplementary Figure 7, and Supplementary Tables 1, 2). In summary, the phosphomimicking mutations do not have major structural effects on the structure of the ARPP proteins.

### ARPP-16 and ARPP19 Interaction Mechanism With PP2A

In order to understand how ARPPs achieve PP2A inhibition, the detailed knowledge of ARPP-PP2A interaction is crucial. As PP2A inhibition is achieved via phosphorylated ARPPs, the binding affinities of both WT and phosphomimicking mutants of ARPPs to PP2A scaffolding A-subunit (PR65α) and different regulatory B56 subunits were first determined using MST, which is based on the directed movement of molecules in a temperature gradient. The A- and B-subunits were used instead of PP2A holoenzyme to learn the role of individual subunits in the ARPP mediated inhibition mechanism. NMR spectroscopy was then applied to map the region in ARPP that interacts with the A-subunit.

**FIGURE 7:**
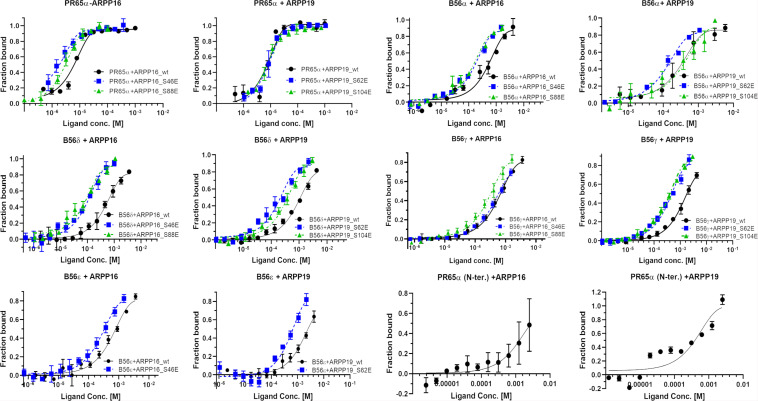
The A-subunit provides a scaffold for ARPPs in PP2A whereas B56 subunits regulate the specificity. The binding curves obtained from MST experiments show that both ARPPs interact strongly with the scaffolding A-subunit. The interaction between WT and phosphomimicking mutants with the A-subunit is similar. The ARPPs interact in different way with various B56 isoforms, and the strength of the interactions is dependent on the phosphorylation state and site. MST measurements were performed using fluorescent labeled A- or B56 subunit as a target and unlabeled ARPP as a ligand.

First, we tested the binding of both ARPP proteins to the scaffolding A-subunit, which has earlier been reported to interact with ARPP-16 (Andrade et al., 2017). Both ARPP proteins were observed to bind with relatively high-affinity to PR65α (A-subunit) as expected, having dissociation constants of 7.9 ± 1.5 μM (ARPP-19) and 5.4 ± 0.7 μM (ARPP-16) (Figure 7 and Table 1.) The N-terminal A-subunit fragment (a.a. 1–274) was also observed to interact with ARPP-16 and ARPP-19, but affinity is significantly weaker than with the full-length A-subunit (Figure 7). Unfortunately, we were not able to express the residual C-terminal fragment of the A-subunit. Interestingly, phosphorylation does not have a major effect on the affinity between the A-subunit and ARPP-19. However, S46E mutation in ARPP-16 increases it’s affinity toward the A-subunit by fivefold in comparison to WT. ARPP-16 S88E mutation, in turn, cause only minor increase on the affinity (Figure 7 and Table 1).

**TABLE 1:** The binding affinities between ARPP-16/19 and PP2A A- and B56-subunits determined using MST.

PP2A subunit	ARPP-19	K_*d*_ ± SEM (μM)	ARPP-16	K_*d*_ ± SEM (μM)
A-subunit	WT	7.9 ± 1.5	WT	5.4 ± 0.7
	S62E	7.5 ± 1.9	S46E	1.0 ± 0.3
	S104E	6.1 ± 1.3	S88E	3.3 ± 0.5
B56α	WT	470 ± 220	WT	80 ± 32
	S62E	110 ± 27	S46E	110 ± 26
	S104E	85 ± 40	S88E	130 ± 34
B56δ	WT	430 ± 100	WT	>700
	S62E	95 ± 25	S46E	110 ± 33
	S104E	195 ± 40	S88E	40.7 ± 9.2
B56γ	WT	>1,300	WT	710 ± 138
	S62E	195 ± 35	S46E	440 ± 120
	S104E	320 ± 40	S88E	200 ± 50
B56ε	WT	>800	WT	>500
	S62E	>700	S46E	250 ± 100
	S104E	No binding detected	S88E	No binding detected

Next, we determined the interactions of both ARPP proteins with four regulatory B56 isoforms, B56α, B56δ, B56γ, and B56ε. Interactions to B56 different subunits were investigated as B56 have crucial tumor suppressor role and the knowledge about ARPP—B56 interactions is scarce (Chen et al., 2004; Arnold and Sears, 2006; Eichhorn et al., 2009). The results show that the affinity of the ARPP proteins to these B56 subunits is much weaker than to the A-subunit (Figure 7 and Table 1). Interestingly, the interactions between ARPP-16/19 and the different B56 subunits are not similar. ARPP-19 WT has the strongest affinity to B56α, while the interaction with the other B56 isoforms is significantly weaker. ARPP-16 WT, interacts weaker with B56α than ARPP-19, whereas the ARPP-16 affinity toward B56γ and B56ε is stronger than with ARPP-19. With the B56δ isoform the situation is, in turn, *vice versa* (Figure 7 and Table 1). In general, the phosphorylation increases the affinity of both ARPP proteins toward almost all B56 subunits, only B56ε-ARPP-16 S88E/ARPP-19 S104E are exceptions, as can be seen from the comparison of the MST curves shown in Figure 7 and Table 1. The site of the phosphorylation has a great influence on the strength of the interaction. ARPP-19 S62E, which corresponds MAST3 and GWL kinases phosphorylated ARPP-19, interacts strongest with B56α and B56δ while it has much lower affinity toward the other B56 isoforms. The interaction pattern of the S104E mutation, corresponding PKA kinase phosphorylated ARPP-19, is different from that of ARPP-19 S62E. Compared to WT, the S104E mutation increases the affinity toward B56δ, and B56γ, and B56α. No interaction with B56ε was observed. The interactions of ARPP-19 S104E with all B56 isoforms are much weaker or similar than those of ARPP-19 S62E (Figure 7 and Table 1). Compared to WT, both ARPP-19 phosphomimicking mutants significantly increase the affinity toward B56δ and B56γ. The phosphomimicking mutations in ARPP-16 have different influence on the binding affinity to the B56 subunits than seen with ARPP-19. Both S46E and S88E, increase the affinity of ARPP-16 toward B56γ and B56δ while the interaction to B56α was similar to that of ARPP16 WT. The S46E also increased the affinity to B56ε, whereas the interaction between ARPP-16 S88E and B56ε is not even observed. To summarize, the specificity of ARPP-16 and ARPP-19 toward different regulatory subunits is different. As expected, the phosphomimicking mutations increase the affinity to the regulatory subunits. The strongest interactions are seen with B56α, but the most considerable effect of the phosphomimicking mutation in the affinity is seen with B56δ, B56γ, and B56ε, except between ARPP-16 S46E-B56γ, where the phosphomimicking mutation has no influence on the interaction.

**FIGURE 8:**
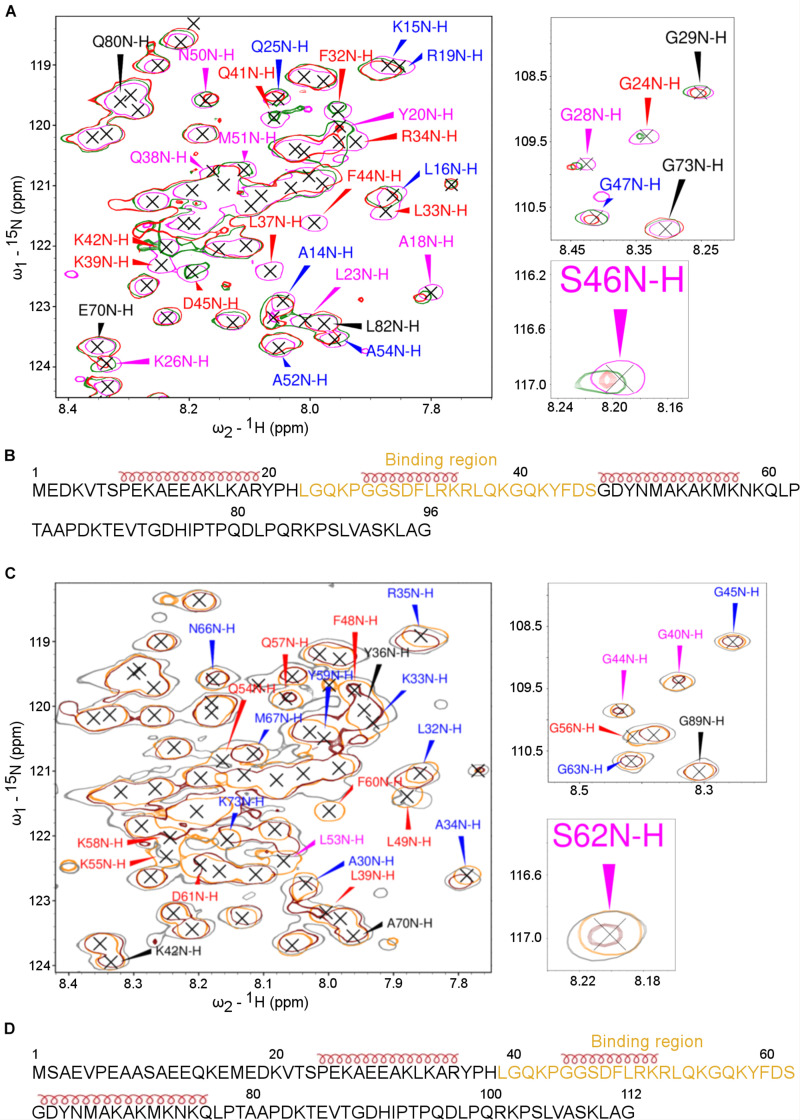
The PP2A A-subunit binds to the 2nd transient α-helices. HSQC spectra of ^15^N-labeled ARPPs collected before and after the addition of the PP2A A-subunit reveals the A-subunit interacts with the region that based on SSP calculations form a transient α-helices. **(A)** The overlaid of ^15^N-HSQC of ^15^N-labeled free ARPP-16 (magenta), 1:1 (green), 1:2 (red) ARPP-16: PP2A A-subunit. The NH cross peaks that broadened with 1:2 PP2A A-subunit-ARPP-16 are labeled red, whereas the cross peaks that shifted the most and exhibit significant line broadening are labeled with magenta and the NH correlations with small CSPs are labeled blue. Those NH cross peaks whose intensity and position remained intact are labeled with black color. **(B)** ARPP-16 sequence showing the epitope that interacts with the A-subunit of PP2A as well as the positions of transient α-helices obtained from SSP calculations are shown. **(C)** The overlaid of ^15^N-HSQC of ^15^N-labelled free ARPP-19 (orange), 1:1 (gray) and 1:2 (maroon) ratio with PP2A A-subunit. The effect to the addition of 1:2 ratio of A-subunit into ARPP-19 are labeled similarly as in panel **(A)**. **(D)** ARPP-16 sequence showing the region that interacts with the A-subunit of PP2A as well as the positions of the transient α-helices obtained from SSP calculations.

The NMR spectroscopy was then applied to characterize further the interaction between the ARPP proteins and the A-subunit. First, the titration of the ^15^N-labeled ARPP-16 WT and ARPP-19 WT with the A-subunit confirmed the interaction between these proteins. However, the overall cross peak distribution in the ^15^N-HSQC spectra of ARPP-16/19 remained similar after the addition of the A-subunit into the free ARPP-16/19 samples (Figures 8A,C). This suggests that no major binding induced structural rearrangement took place upon the addition of the A-subunit, that is ARPP-16/19 remain structurally disordered in the regions flanking the binding motif (*vide supra*). Nevertheless, the closer inspection of the ^15^N-HSQC spectra revealed small but clearly detectable peak shifts for both ARPP proteins, indicating changes in the average chemical environment of the corresponding residues, as expected for the interaction with the A-subunit (Figures 8B,D).

To determine the A-subunit binding sites at ARPP-16/19 at the residue level, we monitored the A-subunit binding induced chemical shift perturbations (CSPs) in the ^15^N-HSQC spectra (Supplementary Figures 8, 9). At 1:1 (ARPP-19: A-subunit) concentration ratio, we observed small CSPs together with a significant line broadening for the following ARPP-19 NH cross-peaks: F48, L49, Q54, K55, G56, K58, and F60. When the A-subunit concentration was increased to 1:2 (ARPP-19: A-subunit), all the above-mentioned NH cross-peaks disappeared and the additional L39 peak disappeared (Figure 8A). The close comparison of the ARPP-19 ^15^N-HSQC spectrum in the absence of the A-subunit to the spectra with one or twofold excess of the A-subunit revealed that the cross peaks corresponding the amino acid residues L39-S62 are either shifted or strongly exchange broadened already at 1:1 ratio, and the effect is increased with 1:2 ratio (Figure 8A). Thus, based on these results, the A-subunit interacting region in ARPP-19 corresponds to residues L39-S62. This sequence consists of the residues of the second transient α-helix as well the flanking disordered regions at both sides (Figure 8B).

In the next phase, we investigated the A-subunit binding to ARPP-16. Comparison of the ^15^N-HSQC ARPP-16 spectra measured with ARPP-16: PP2A A-subunit concentration ratios of 1:1 and 1:2 shows that the A-subunit binding regions in ARPP-16 and ARPP-19 are identical (Figures 8B,D). Indeed, the A-subunit interacts with residues L23-S46 in ARPP-16, which is similar as with ARPP-19, corresponding the second transient α-helix and the flanking disordered regions at both sides (Figures 8A,C). Similar to ARPP-19, the PP2A A-subunit binding induced severe line broadening in ARPP-16. More specifically, at ARPP-16: PP2A A-subunit ratio 1:1, we observed the disappearance of the following ARPP-16 NH resonances: L33, R34, K35, L37, Q38, K39, and F44. When the A-subunit concentration was further increased to 1:2 ratio (ARPP-16: -A-subunit), also L23, G24, F32, Q41, K42, and D45 cross-peaks disappeared. These NMR titration data are in good agreement with our results obtained from the MST binding assay. Indeed, the K_d_ value in the range of 5 to 10 μM typically results in significant line broadening of NMR resonances at the binding interface due to intermediate exchange limit on NMR timescale. However, the regions in ARPP-16/19 flanking the A-subunit binding motif remain highly mobile and sharp resonances can be observed for these residues also in the bound state. This suggests that the ARPP-16/19-A-subunit interaction is mediated through a linear motif established by the residues in the second transient helix and more disordered residues between the helices two and three. Unfortunately, we could not purify B56 proteins in the concentrations needed for the NMR titration experiments, and therefore it was not possible to map the B56 binding sites in ARPP-16 and ARPP-19.

## Discussion

The inhibition of the PP2A activity has been shown to promote malignant transformation in human cancer cells as PP2A inhibition results in hyperphosphorylation of large number of oncogenic drivers. In cancer cells, PP2A is inhibited by numerous otherwise unrelated proteins, called cancerous PP2A inhibitor proteins, which are overexpressed in a wide range of cancer types (Meeusen and Janssens, 2018). ARRP-16/19 proteins have become the focus of many studies in the past few years as a result of their discovery as potent inhibitors of PP2A (Gharbi-Ayachi et al., 2010; Mochida et al., 2010), and their role in several cancer types such as HCC (Song et al., 2014), human glioma (Jiang et al., 2016), and AML (Mäkelä et al., 2019). Despite progressive advances in our understanding of the molecular biology of these diseases, the outcome for most patients is still poor. It is, therefore, necessary to develop more effective treatment strategies. It is a therapeutically tempting idea to restore the PP2A activity by preventing the PP2A—PP2A inhibitor protein interaction by small molecules. The fact the PP2A complex is mutated at a relatively low frequency in most human cancer cells provides bases to this strategy (Arroyo and Hahn, 2005; Kauko and Westermarck, 2018). Furthermore, the recent reports of small molecules and peptides that are capable of restoring the PP2A activity in human cancer cell lines provide convincing support to this strategy to be used as cancer therapeutics (Perrotti and Neviani, 2013; Farrell et al., 2014). To develop small molecules that prevent the PP2A inhibitor proteins binding to PP2A, it is crucial to understand the structural and molecular level PP2A inhibition mechanism.

Here, we have characterized the structural properties of the PP2A inhibitor protein ARPP-19 and its splice variant ARPP-16 as well as their interaction with the different PP2A subunits. ARPP-16/19 establish a structurally new class of cancerous PP2A inhibitor proteins. Unlike all other thus far known PP2A inhibitor proteins such as CIP2A (Wang et al., 2017) and PME-1 (Xing et al., 2008), based on the results presented here, ARPP-16 and ARPP-19 lack a well-defined 3D structure. This is in accordance with earlier study where ARPP-19 was reported having a random coil conformation (Huang et al., 2001). Usually, IDPs do not exhibit fully random coil behavior, but adopt transiently populated secondary structures (Mollica et al., 2016). This is also the case with both ARPP proteins which have few regions that have the propensity to form transient α-helical structures, whereas the terminal residues of both proteins are very mobile. The structural ensemble of ARPP-16/19, built using the experimental restraints from NMR and SAXS data, revealed that the ensemble of both ARPP proteins are characterized by a combination of compact and extended conformations, and extended conformers are suggested by EOM to comprise a minor subset of structures. The presence of highly extended sub-population of WT ARPPs might be the result of charge-mediated repulsion because the expansion of dimension clearly correlates to the net charge of the protein and ARPP-19 have a net charge of +3 and ARPP16 have a slight excess of positive charge (net charge + 7). The introduction of phosphomimetic mutation Ser-to-Glu decreases the net charge of the ARPPs and lead to the coil to globule transition (Mao et al., 2010; Muller-Spath et al., 2010; Testa et al., 2013). On the other hand, it is also possible that the introduction of S46E/S62E mutation in ARPP-16/19 leads to the more extended conformation due to charge-charge repulsion as it is in close vicinity to the negatively charged residues D45/D61 and D48/D64. According to our EOM result, the more extended conformation is not present in these mutants. The charge-charge repulsion of the mutated glutamate residue and the nearby aspartate residue might have pushed each other away that allowed them to make charge-charge interaction with the nearby ionizable groups, R34/R50, K35/K51, R36/R52, K39/K55, K42/K58 to stabilize compact structural state. Yet the phosphomimetic mutation S88E/S104E in ARPP-16/19 might stabilize structure by charge-charge interaction with nearby ionizable group, R85/R101, K86/K102, and K93/K109 and drive nascent of more compact conformational ensemble. The phosphorylation sites at ARPP-16/19 are located outside of the transiently populated helical segments, which is often the case with IDPs because the disordered regions are easily accessed by modifying enzymes for post-translational modifications (Iakoucheva et al., 2004; Mattinen et al., 2006; Dyson, 2016). Neither of the phosphorylation sites increases the structural order in ARPP-16/19, but interestingly the phosphomimicking mutants of ARPP-16/19 decrease the population of the most extended conformers in comparison to the WT ARPP proteins.

The lack of a stable 3D structure has an advantage in the regulation of the intermolecular interactions between binding partners. The nature of intermolecular interactions between IDPs and their folded binding partner is usually highly dynamic with rapid association and dissociation, having high specificity but low affinity because of decrease in conformational entropy upon binding, and facilitating rapid exchange of binding sites between multiple interacting partners (Mészáros et al., 2007; Zhou, 2012; Berlow et al., 2015; Dyson, 2016; Mollica et al., 2016; Schuler et al., 2020). Our results from PP2A—ARPP interaction assay reveal that both ARPP proteins interact strongly, with low μM affinity, with the scaffolding A-subunit. This is in accordance with the earlier study by Andrade et al. (2017), which reported that ARPP-16 directly binds to A-subunit. The A-subunit binding sites at ARPP-16/19 were mapped using NMR spectroscopy into the motif formed by the second transient α-helix and the flanking amino acids. Such structural pre-organization has often been found relevant in the intermolecular interactions undergone by IDPs (Wright and Dyson, 2015). While some cases IDPs may fold to complete structured conformation upon binding, often the binding of IDPs to physiological partners is accompanied by the gain of structure only in the binding region (Berlow et al., 2015; Wright and Dyson, 2015; Schuler et al., 2020; Toto et al., 2020). This is also the case with ARPP—A-subunit interaction, where the binding induced CSPs along with the line broadening can be readily observed for the residues at the binding region. Regions flanking the binding motif maintain high degree of disorder upon binding. The phosphomimicking mutations do not either have any major influence on the strength of ARPP—A-subunit interaction, suggesting the binding is mediated through a short linear motif. This is common feature of IDPs which often recognize the binding partners through short linear motifs and regions outside of these binding motifs remain largely disordered, leading to the formation of so called fuzzy complexes (Berlow et al., 2015; Schuler et al., 2020). The complexes between IDPs and their folded binding partners are usually highly dynamic where the IDP stays almost completely disordered and forms multivalent, rapidly exchanging interactions involving only transient local ordering (Schuler et al., 2020).

The interactions of ARPP-16/19 with the regulatory B-subunits are significantly weaker than with the A-subunit. Generally, the phosphorylation of ARPP-16/19 increases the affinity toward all regulatory B56 subunits. Unlike with the scaffolding subunit, the affinity of ARPP-16/19 toward different regulatory subunits is different depending on the state and site of phosphorylation. In summary, based on our results, the A-subunit provides a scaffold for ARPP—PP2A interaction, and then the specificity of PP2A inhibition is achieved via the phosphorylated ARPP—regulatory subunit interaction. Most likely ARPP-16/19 have two distinct binding sites, one for the scaffolding subunit, and another for the regulatory subunit. The ability to bind their targets through multiple sites, which do not function independently but synergistic coupling between independent binding sites is common feature of IDPs (Berlow et al., 2015; Wright and Dyson, 2015).

Interestingly, the B-subunit interacts with HEAT repeats 2–8 of the A-subunit (Cho and Xu, 2007), which based on Andrade et al. (2017) is the same region where ARPP-16 binds. Accordingly, the strong ARPP-16/19 interaction with the A-subunit, might act as an assembly base to escort ARPP proteins and facilitate interaction with B-subunit of the holoenzyme and PP2A inhibition. Interestingly, the affinity of ARPP-19 and ARPP-16 toward to the scaffolding A- and regulatory B-subunits are not similar although their amino acid sequences are otherwise similar but ARPP-19 has extra 16 amino acid residues at the N-terminus. The mapped A-subunit binding sites at the both ARPP proteins are, however, the corresponding regions. Accordingly, it seems that the N-terminal tail in ARPP-19 interferes with the A- and B-subunit binding epitopes in ARPP-19. However, the complete picture about PP2A—ARPP interaction mechanism remains elusive as the interaction studies have been performed with PP2A subunits not with PP2A holoenzyme.

The function of PP2A is greatly influenced by its holoenzyme assembly. The variability in PP2A holoenzyme composition results in an amazingly diverse enzyme with vast array of substrate specificities. The knowledge about the selectivity of ARPP-16/19 toward different regulatory subunits is still scarce. Here we concentrated on the B56 subunits due to their crucial role in tumor suppression (Chen et al., 2004; Li et al., 2007; Nobumori et al., 2013). MAST3 kinase phosphorylated ARPP-16 has been reported to selectively inhibit PP2A holoenzyme containing B55α and B56δ (Andrade et al., 2017). In the same manner, our results show that ARPP-16 S46E and ARPP-19 S62E, which corresponds MAST3 kinase phosphorylated ARPP-16 and ARPP-19, bind strongest to B56δ and B56α. However, on should bear in mind that one of the limitations of this study is the use of serine-to-glutamate mutation to investigate the effect of phosphorylation. The chemical properties of the carboxylic group of glutamate differ from that of phosphorylated serine, like the charged state, size, and geometry (Chen and Cole, 2015). Therefore, the Ser-to-Glu mutation may not be sufficient to study the effect of phosphorylation, as it cannot always restore the function of phosphorylation.

There is growing interest in IDPs as potential targets for drug design (Cheng et al., 2006). IDPs usually bind with modest affinity, like here ARPP-16/19 with PP2A subunits. IDPs bind usually into concave grooves in the surface of their targets predominantly through hydrophobic interactions (Mészáros et al., 2007), making them very attractive therapeutic targets. Researchers have tried to mimic IDP-target protein interaction by synthesizing conformationally constrained molecules to inhibit IDP—target protein interaction (Lao et al., 2014a, b). There are also recent reports where the protein-protein inhibitor molecule has successfully been targeted to bind the IDP rather than its globular target (Krishnan et al., 2014; Vendruscolo et al., 2015; Neira et al., 2017). Accordingly, there is a proven consent for targeting ARPP-19 to prevent ARPP-19—PP2A interaction to treat ARPP-19 related cancer types.

The inhibition of PP2A complexes function by the overexpression of PP2A inhibitor proteins is one of the most important reasons for the transformation of normal cells into malignant cells. Accordingly, the understanding of the molecular and structural bases of PP2A inhibition is crucial for the development of new therapeutics for cancer. Our results show the PP2A inhibitor protein ARPP-19, and its splicing variant ARPP-16, do not form a well-defined 3D structure but are intrinsically disordered. Both ARPP-16 and ARPP-19 apply the two-state mechanism on the PP2A interaction; the PP2A A-subunit is used as a scaffold to establish the interaction while the B56 subunit regulates the specificity. Although our results do not give a complete molecular and structural level explanation for the ARPP mediated PP2A inhibition, our results provide a good starting point.

## Data Availability Statement

The chemical shifts of ARPP-16 and ARPP-19 have been deposited to Biological Magnetic Resonance Data Bank under the accession numbers 27911 and 27912, respectively. All other data supporting the conclusions of this article are included within the article and its supporting information.

## Author Contributions

CT, PR, and TH cloned, expressed, and purified the constructs. CT performed NMR and SAXS experiments and their analyses. CT and PR performed MST measurements. All authors designed experiments and contributed the manuscript writing.

## Conflict of Interest

The authors declare that the research was conducted in the absence of any commercial or financial relationships that could be construed as a potential conflict of interest.
