# Supplementary material for: The Interaction Mechanism of Intrinsically Disordered PP2A Inhibitor Proteins ARPP-16 and ARPP-19 With PP2A
Source: Front Mol Biosci. 2021 Mar 26;8:650881. doi: 10.3389/fmolb.2021.650881 (PMC8032985; doi:10.3389/fmolb.2021.650881)
Supplement: Supplementary file 1 [file Table_1.DOCX]

Supplementary Material


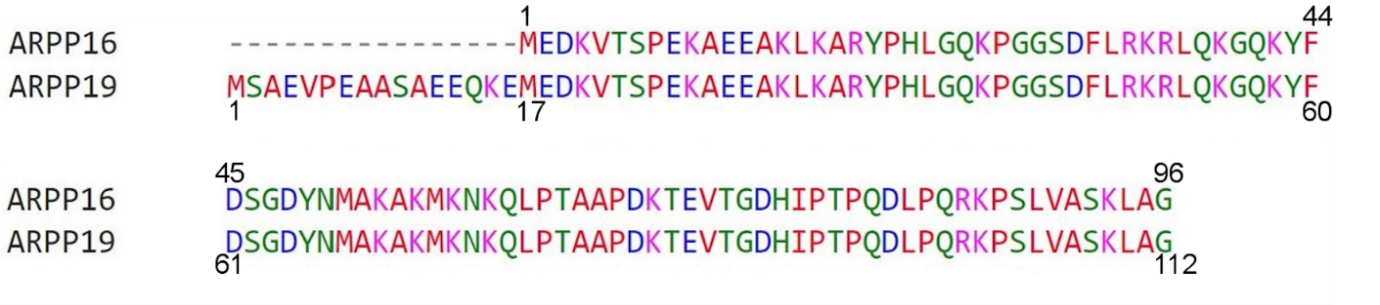


**Supplementary Figure 1.** The sequence alignment of ARPP-19 and ARPP-16.

**
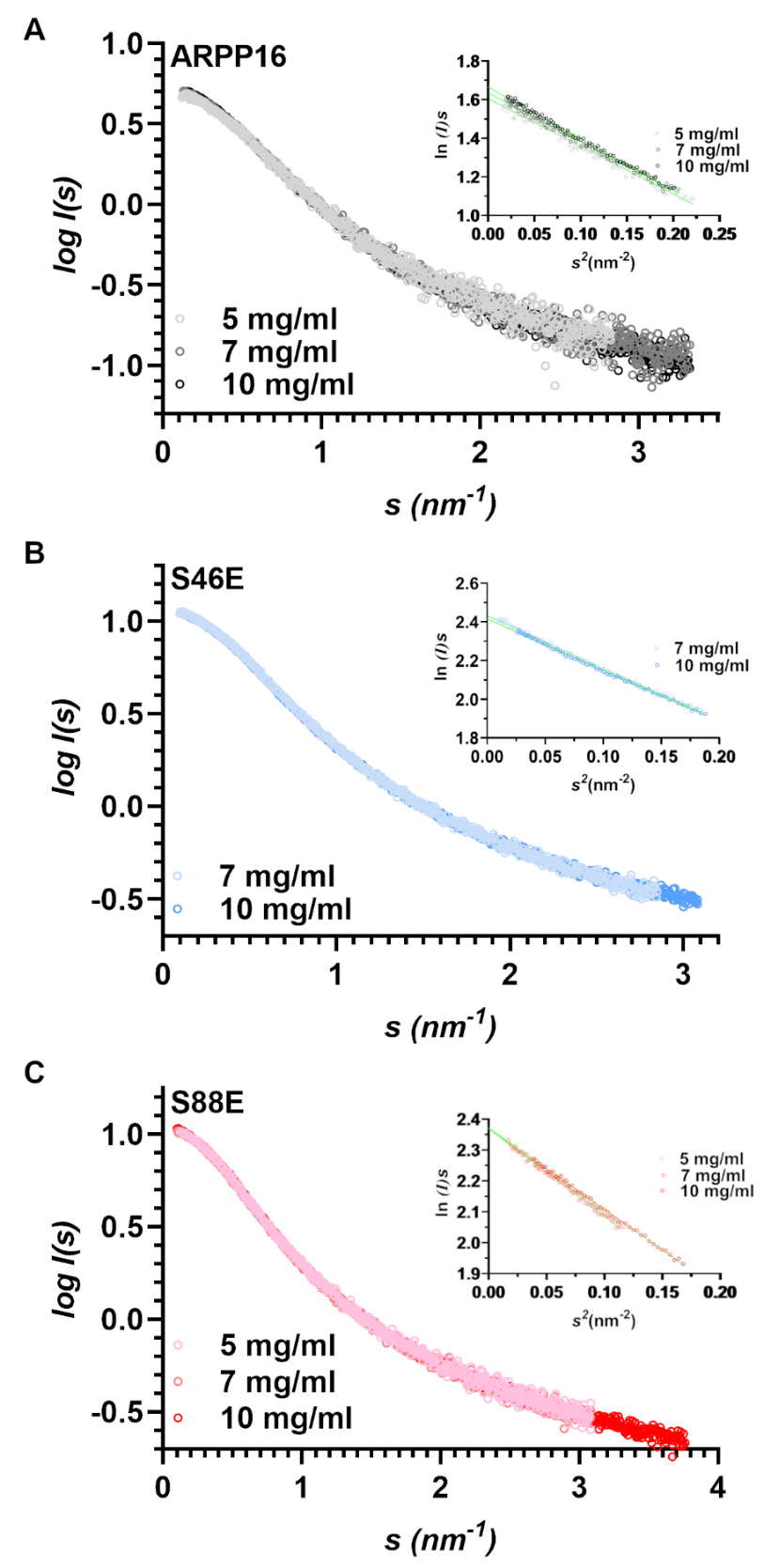
**

**Supplementary Figure 2.** Primary small angle X-ray scattering (SAXS) analysis of ARPP-16 and its phosphomimicking mutants related to Figure 4 and 5 in this study. The solution scattering profiles with the insets showing the Guinier fits of (A) ARPP-16, (B) ARPP-16 S46E and (C) ARPP-16 S88E at 5, 7 and 10 mg/ml concentrations. The IDPs exhibit multiple conformations and display a different scattering profile. Therefore, the resulting scattering curves of IDPs are smoothened upon averaging and lack specific features. The scattering profile of ARPP-16 S46E at 5mg/ml concentration is missing due to the error in data acquisition.

**
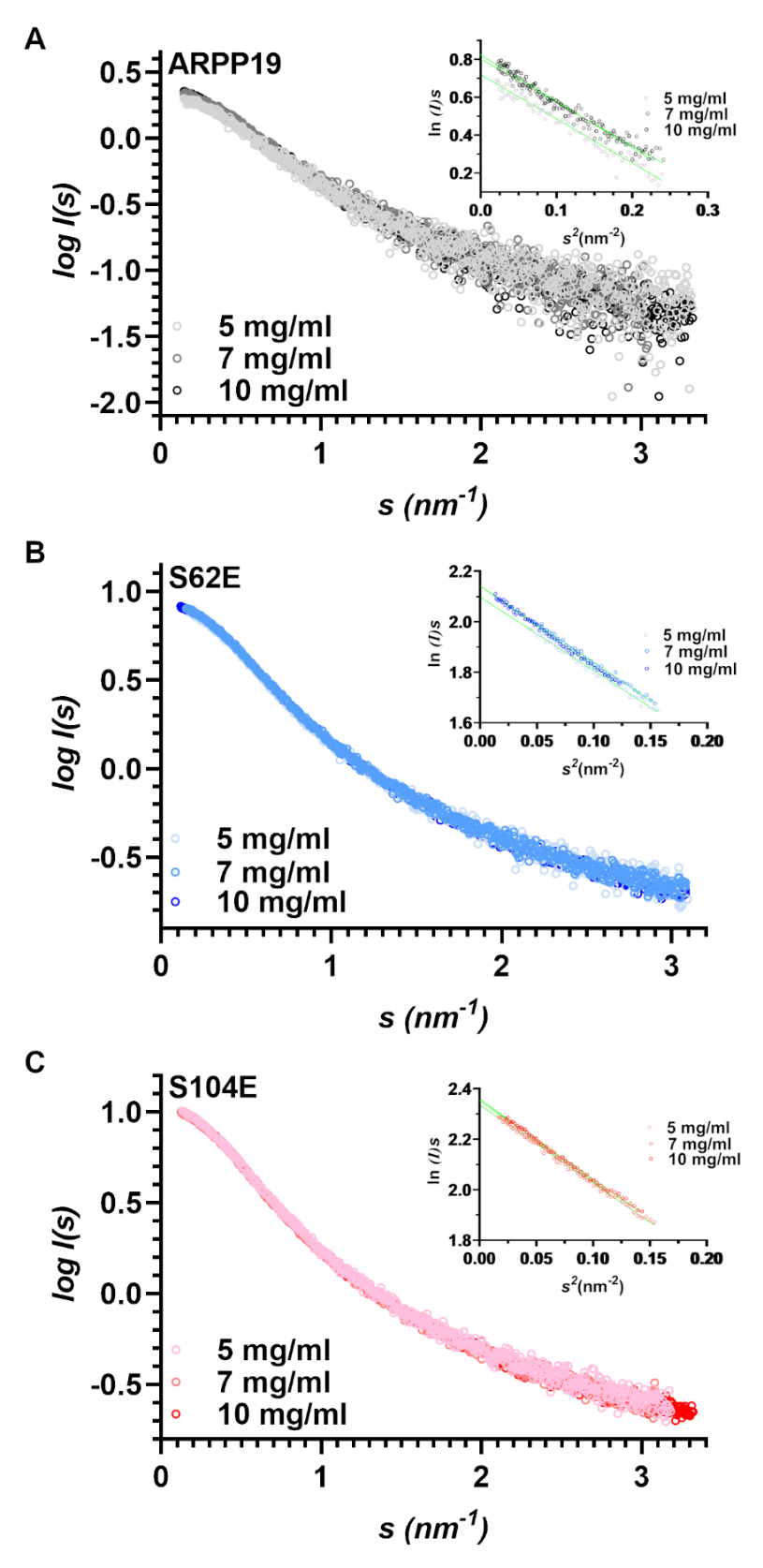
**

**Supplementary Figure 3.** Primary small angle X-ray scattering (SAXS) analysis of ARPP-19 and its phosphomimicking mutants related to Figure 4 and 5 in this study. The solution scattering profiles with the insets showing the Guinier fits of (A) ARPP-19, (B) ARPP-19 S62E and (C) ARPP-19 S62E at 5, 7 and 10 mg/ml concentrations.

**Supplementary Table 1.** Primary SAXS data processing of the ARPP-16 proteins and their phosphomimicking mutants related to Figure 4 in this study.

|  |  | **ARPP-16** | | |  | **ARPP-16 S46E** | | |  | **ARPP-16 S88E** | | |
| --- | --- | --- | --- | --- | --- | --- | --- | --- | --- | --- | --- | --- |
|  |  | **5 mg/ml** | **7 mg/ml** | **10 mg/ml** |  | **5 mg/ml** | **7 mg/ml** | **10 mg/ml** |  | **5 mg/ml** | **7 mg/ml** | **10 mg/ml** |
|  |  |  |  |  |  |  |  |  |  |  |  |  |
| ***Guinier analysis*** | |  |  |  |  |  |  |  |  |  |  |  |
|  | *I*(0) (arbitary units) | 5.0 ± 0.02 | 5.1 ± 0.02 | 5.3 ± 0.01 |  |  | 11.35 ± 0.02 | 11.19 ± 0.02 |  | 10.74 ± 0.04 | 10.68 ± 0.06 | 10.7 |
|  | *R*_g_ (nm)^a^ | 2.74 ± 0.02 | 2.76 ± 0.01 | 2.84 ± 0.02 |  |  | 2.85 ± 0.05 | 2.83 ± 0.06 |  | 2.89 ± 0.02 | 2.92 ± 0.02 | 2.81± 0.01 |
|  | s_min_ (nm^-1^) | 0.352 | 0.38 | 0.42 |  |  | 0.27 | 0.53 |  | 0.44 | 0.38 | 0.56 |
|  | s*R*_g_ max (nm^-1^) | 1.29 | 1.25 | 1.27 |  |  | 1.22 | 1.23 |  | 1 | 0.98 | 1.15 |
|  | Coefficient of correlation, *R*^2^ | 0.987 | 0.991 | 0.996 |  |  | 0.996 | 0.998 |  | 0.991 | 0.995 | 0.998 |
|  |  |  |  |  |  |  |  |  |  |  |  |  |
| ***P(r) analysis*** | |  |  |  |  |  |  |  |  |  |  |  |
|  | *I*(0) (arbitary units) | 5.1 ± 0.02 | 5.2 ± 0.02 | 5.4 ± 0.02 |  |  | 11.5 ± 0.02 | 11.3 ± 0.02 |  | 10.79 ± 0.03 | 10.71 ± 0.02 | 10.89 ± 0.02 |
|  | *R*_g_ (nm) | 2.9 ± 0.02 | 2.9 ± 0.02 | 3.03 ± 0.02 |  |  | 3.0 ± 0.01 | 3.0 ± 0.01 |  | 3.0 ± 0.02 | 3.0 ± 0.01 | 2.9 ± 0.01 |
|  | D_max_ (nm)^b^ | 10.7 | 11.2 | 11.85 |  |  | 11.45 | 11.85 |  | 11.6 | 12 | 11.7 |
|  | s range (nm^-1^) | 0.124 - 2.824 | 0.138 -2.889 | 0.129 - 2.810 |  |  | 0.096 - 2.805 | 0.162 - 2.918 |  | 0.129 - 2.862 | 0.110 - 2.542 | 0.1521 - 2.815 |
|  | *X*^2^ (total estimate from GNOM) | 0.95 (0.79) | 0.98 (0.78) | 0.87 (0.75) |  |  | 0.92 (0.75) | 0.79 (0.72) |  | 0.93 (0.76) | 0.80 (0.73) | 0.92 (0.76) |
|  | |  |  |  |  |  |  |  |  |  |  |  |

**Supplementary Table 2.** Primary SAXS data processing of the ARPP-19 proteins and their phosphomimicking mutants related to Figure 4 in this study.

|  |  | **ARPP-19** | | |  | **ARPP-19 S62E** | | |  | **ARPP-19 S104E** | | |
| --- | --- | --- | --- | --- | --- | --- | --- | --- | --- | --- | --- | --- |
|  |  | **5 mg/ml** | **7 mg/ml** | **10 mg/ml** |  | **5 mg/ml** | **7 mg/ml** | **10 mg/ml** |  | **5 mg/ml** | **7 mg/ml** | **10 mg/ml** |
| ***Guinier analysis*** | |  |  |  |  |  |  |  |  |  |  |  |
|  | *I*(0) (arbitary units) | 2.1 ± 0.01 | 2.2 ± 0.02 | 2.3 ± 0.01 |  | 8.2 ± 0.04 | 8.5 ± 0.03 | 8.5 ± 0.02 |  | 10.6 ± 0.05 | 10.3 ± 0.02 | 10.5 ± 0.02 |
|  | *R*_g_ (nm)^a^ | 2.64 ± 0.05 | 2.63 ± 0.05 | 2.73 ± 0.04 |  | 2.96 ± 0.03 | 3.01 ± 0.02 | 3.09 ± 0.01 |  | 3.14 ± 0.03 | 3.05 ± 0.02 | 3.05± 0.01 |
|  | s_min_ (nm^-1^) | 0.39 | 0.39 | 0.43 |  | 0.62 | 0.63 | 0.35 |  | 0.6 | 0.39 | 0.46 |
|  | s*R*_g_ max (nm^-1^) | 1.29 | 1.29 | 1.3 |  | 1.17 | 1.18 | 1.08 |  | 1.07 | 1.19 | 1.15 |
|  | Coefficient of correlation, *R*^2^ | 0.957 | 0.954 | 0.973 |  | 0.992 | 0.995 | 0.997 |  | 0.993 | 0.996 | 0.997 |
|  |  |  |  |  |  |  |  |  |  |  |  |  |
| ***P(r) analysis*** | |  |  |  |  |  |  |  |  |  |  |  |
|  | *I*(0) (arbitary units) | 2.1 ± 0.02 | 2.3 ± 0.02 | 2.4 ± 0.02 |  | 8.3 ± 0.04 | 8.6 ± 0.03 | 8.5 ± 0.02 |  | 10.6 ± 0.03 | 10.42 ± 0.03 | 10.58 ± 0.02 |
|  | *R*_g_ (nm) | 2.85 ± 0.06 | 2.85 ± 0.05 | 3.02 ± 0.04 |  | 3.13 ± 0.03 | 3.19 ± 0.2 | 3.19 ± 0.2 |  | 3.2 ± 0.03 | 3.2 ± 0.02 | 3.2 ± 0.02 |
|  | D_max_ (nm)^b^ | 10.9 | 11.2 | 12.2 |  | 12.25 | 12.45 | 12.69 |  | 12.65 | 11.95 | 12.65 |
|  | s range (nm^-1^) | 0.147 - 2.725 | 0.147 - 2.961 | 0.157 - 2.927 |  | 0.208 - 2.749 | 0.162 - 2.589 | 0.115 - 2.570 |  | 0.1521 - 2.622 | 0.129 - 2.622 | 0.1521 - 2.815 |
|  | *X*^2^ (total estimate from GNOM) | 0.75 (0.68) | 0.83 (0.75) | 0.84 (0.72) |  | 0.89 (0.77) | 0.84 (0.74) | 0.79 (0.74) |  | 0.89 (0.63) | 0.72 (0.68) | 0.77 (0.75) |
|  | |  |  |  |  |  |  |  |  |  |  |  |
|  | ^a^ Estimated from Guinier analysis in PRIMUS (Konarev et al., 2003) | | | | | | | | | | | |
|  | ^b^ Calculated using DATGNOM (Petoukhov et al., 2007) | | | | | | | | | | | |


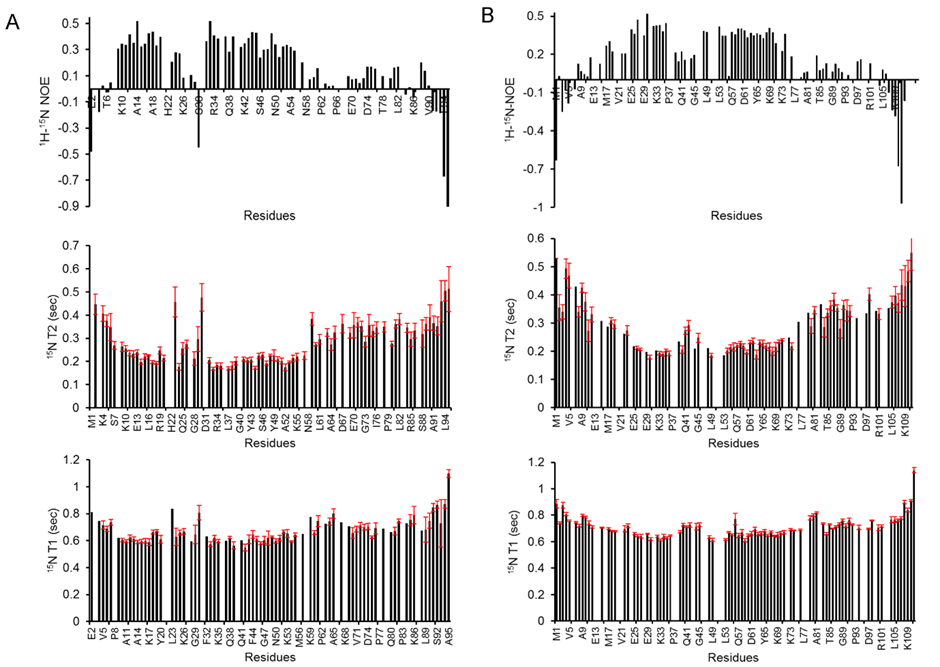


**Supplementary Figure 4. Nuclear spin relaxation times ^15^N T_1_ and ^15^N T_2_ as well as heteronuclear {^1^H}-^15^N NOEs for ARPP-16 and ARPP-19.** (a) ARPP16 and (b) ARPP19**.** The very N-terminal and the C-terminal segment of the ARPPs displayed very low or negative {^1^H}-^15^N NOEs and high transverse relaxation times (^15^N T_2_ values), reflecting the high degree of flexibility in these regions. Few segments in the core region of ARPPs have elevated {^1^H}-^15^N NOE values and decreased ^15^N T_2_ values, that confirms the presence of transient α-helices as indicated by secondary chemical shift analysis.

**
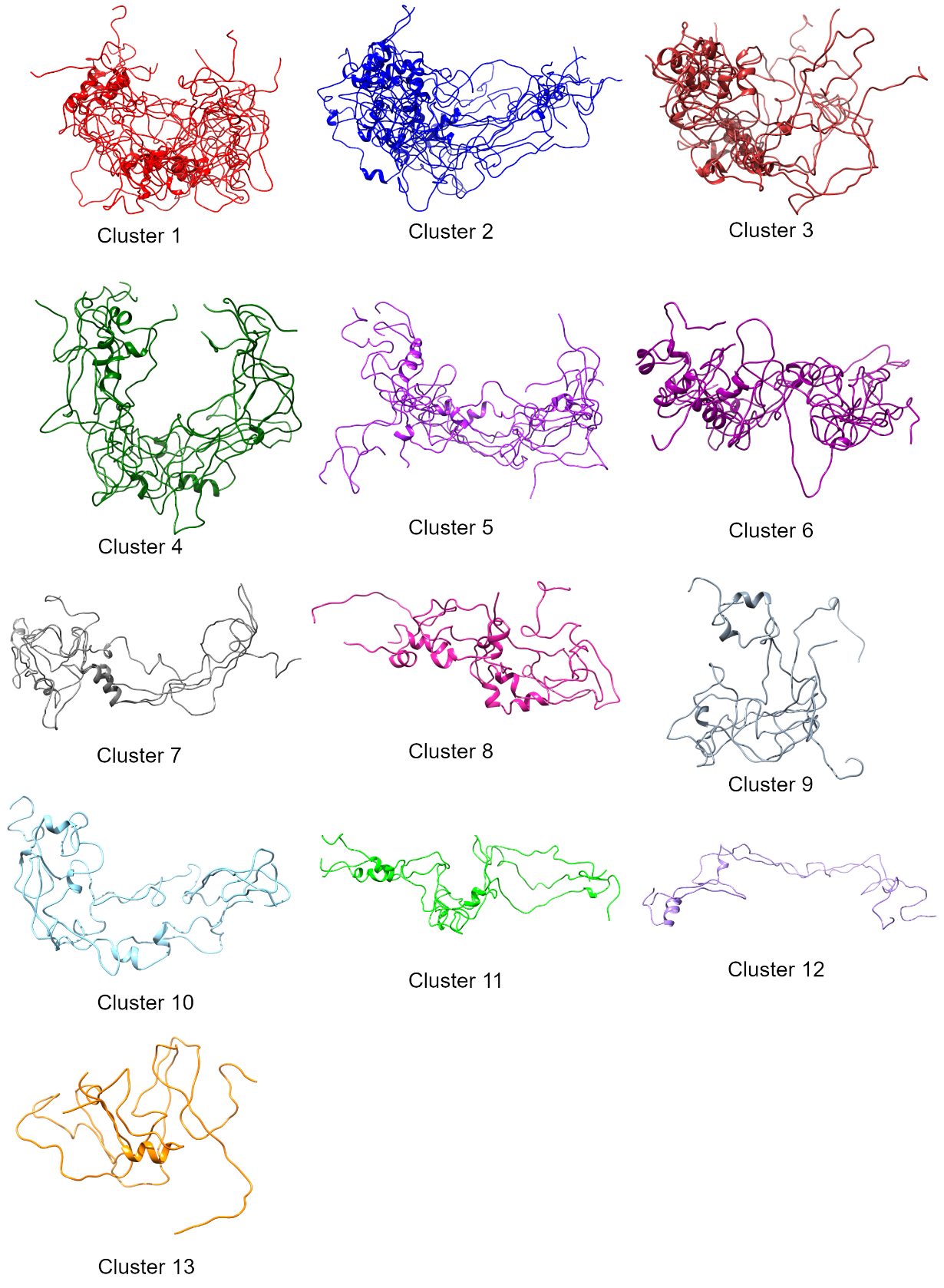
**

**Supplementary Figure 5.** Cluster of substates in the representative ensemble NMR structure of ARPP-16. The top 13 clusters of the representative NMR structure of the total 100 structures are shown.

**
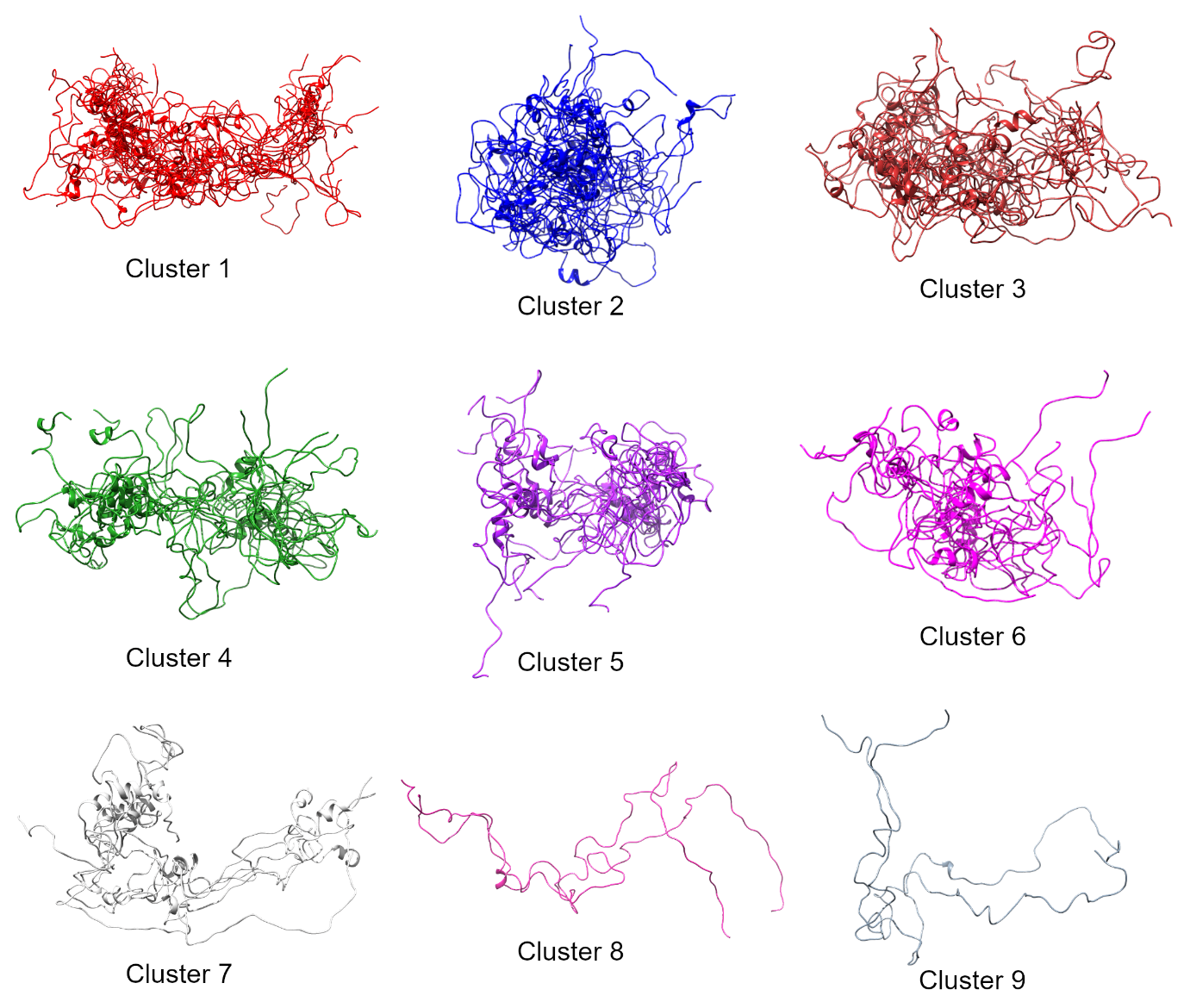
**

**Supplementary Figure 6.** Cluster of substates in the representative ensemble NMR structure of ARPP-19. The top 9 clusters of the representative NMR structure of the total 100 structures are shown.

**Supplementary Table 3.** Cluster properties of ARPP-16 ensemble.

| **Cluster** | **No. of conformers** | **Cα-Cα RMSD** |
| --- | --- | --- |
| 100 ARPP-16 conformers | | |
| 1 | 12 | 18.1 |
| 2 | 15 | 22.9 |
| 3 | 12 | 21.4 |
| 4 | 8 | 18.1 |
| 5 | 9 | 19.2 |
| 6 | 8 | 17.7 |
| 7 | 4 | 18.2 |
| 8 | 3 | 15.8 |
| 9 | 3 | 26.5 |
| 10 | 3 | 17.1 |
| 11 | 3 | 14.2 |
| 12 | 3 | 15.6 |
| 13 | 3 | 19.3 |
| 14 | 1 | 0 |
| 15 | 1 | 0 |
| 16 | 1 | 0 |
| 17 | 3 | 0 |
| 18 | 3 | 0 |
| 19 | 1 | 0 |
| 20 | 3 | 0 |
| Total | 100 | - |

**Supplementary Table 4.** Cluster properties of ARPP-19 ensemble.

| **Cluster** | **No. of conformers** | **Cα-Cα RMSD** |
| --- | --- | --- |
| 100 ARPP-19 conformers | | |
| 1 | 20 | 23.0 |
| 2 | 18 | 22.0 |
| 3 | 17 | 26.4 |
| 4 | 11 | 20.6 |
| 5 | 9 | 19.8 |
| 6 | 8 | 21.7 |
| 7 | 8 | 20.3 |
| 8 | 3 | 18.8 |
| 9 | 2 | 23.6 |
| 10 | 1 | 0 |
| 11 | 1 | 0 |
| 12 | 1 | 0 |
| 13 | 1 | 0 |
| Total | 100 | - |


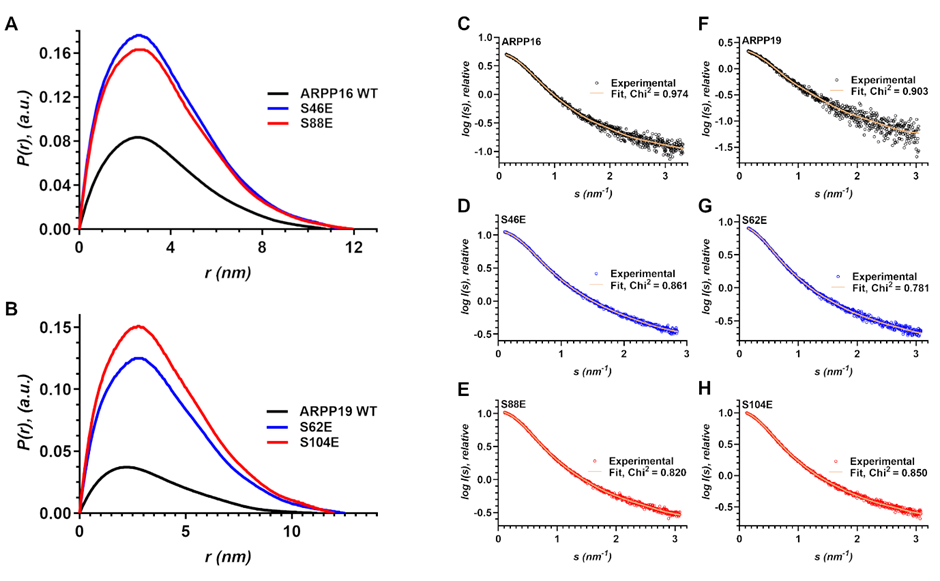


**Supplementary Figure 7.** Pairwise distance distribution, *P(r)*, estimation based on in solution SAXS data and EOM profile fitting for ARPP-16 and ARPP-19 constructs related to figure 4 and 5: (A) pairwise distance distributions function, *P(r)*, derived from ARPP-16 (black), ARPP-16 S46E (blue) and ARPP-16 S88E (red), and (B) pairwise interatomic distance distributions, p(r), derived from ARPP-19 (black), ARPP-19 S62E (blue) and ARPP-19 S104E (red). Typical fits obtained from the selected ensemble of structures to experimental scattering of (C) ARPP-16, (D) ARPP-16 S46E, (E) ARPP-16 S88E, (F) ARPP-19, (G) ARPP-19 S62E and (H) ARPP-19 S104E.

**Supplementary Table 5.** Quantitative EOM analyses of the SAXS data of ARPP proteins and their phosphomimicking mutants related to Figure 4 in this study. All the final ensembles give comparable Rg and Dmax values with the primary data processing of the studied proteins.

|  |  | **ARPP-16^a^** | **ARPP-16 S46E^a^** | **ARPP-16 S88E^a^** |
| --- | --- | --- | --- | --- |
| ***EOM*^b^** | |  |  |  |
|  | *s* range for fitting (nm^-1^) | 0.129 - 3.317 | 0.096 - 2.848 | 0.105 - 3.082 |
|  | Type of models generated | random coil | random coil | random coil |
|  | *X^2^*, *CORMAP P-value* | 0.974, 0.484 | 0.861, 0.247 | 0.820, 0.461 |
|  | Constant subtracted | 0.044 | 0.045 | 0.085 |
|  | No. of representative structures | 9 | 10 | 10 |
|  | R_flex_ (random) / R_sigma_ | 85.25 % (85.89 %) /1.04 | 84.6 % (85.7 %) / 0.98 | 84.9 % (86.5%) / 0.93 |
|  | Final ensemble R_g_/D_max_ (nm) | 2.9/8.9 | 3.0/9.3 | 3.0/9.4 |
|  |  | | | |
|  |  | **ARPP-19^a^** | **ARPP-19 S62E^a^** | **ARPP-19 S104E^a^** |
| ***EOM*^b^** | |  |  |  |
|  | *s* range for fitting (nm^-1^) | 0.147 - 3.044 | 0.147 - 3.082 | 0.124 - 3.802 |
|  | Type of models generated | random coil | random coil | random coil |
|  | *X^2^*, *CORMAP P-value* | 0.903, 0.702 | 0.781, 0.994 | 0.850, 0.459 |
|  | Constant subtracted | 0.004 | 0.013 | 0.03 |
|  | No. of representative structures | 6 | 10 | 10 |
|  | R_flex_ (random) / R_sigma_ | 77.4 % (82.99 %) /0.90 | 81.8 % (84.1 %) / 0.94 | 83.9 % (85.6%) / 0.96 |
|  | Final ensemble R_g_/D_max_ (nm) | 2.9/9.4 | 3.2/9.8 | 3.3/10.4 |
|  |  |  |  |  |
|  | ^a^ Experimental scattering data from 7 mg/ml used | | | |
|  | ^b^ https://www.embl-hamburg.de/biosaxs/atsas-online/eom.php (Bernadó et al., 2007; Tria et al., 2015) | | | |
|  | Default parameters, 10 000 models in the initial ensemble. | | |  |

**
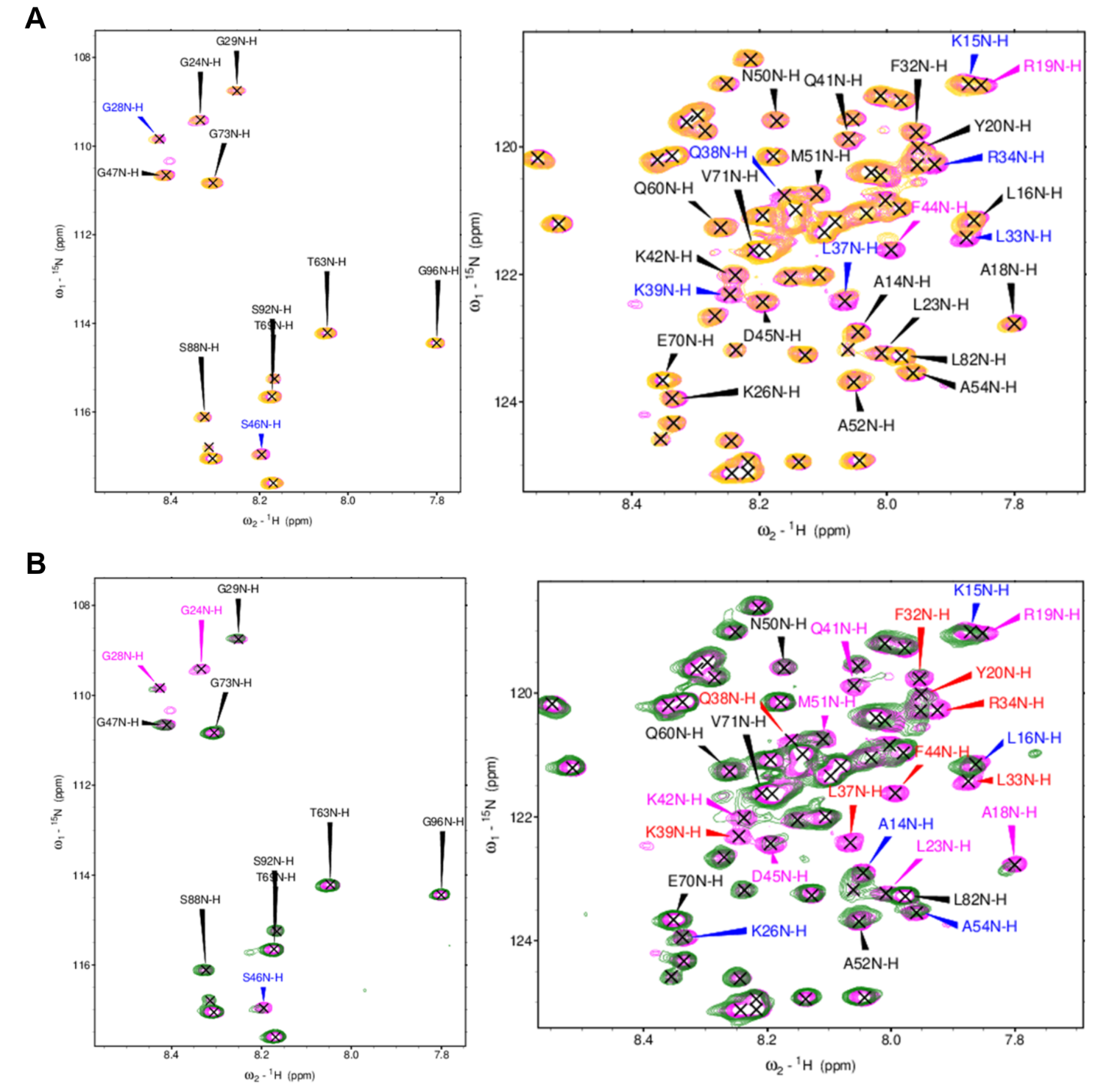
**

**
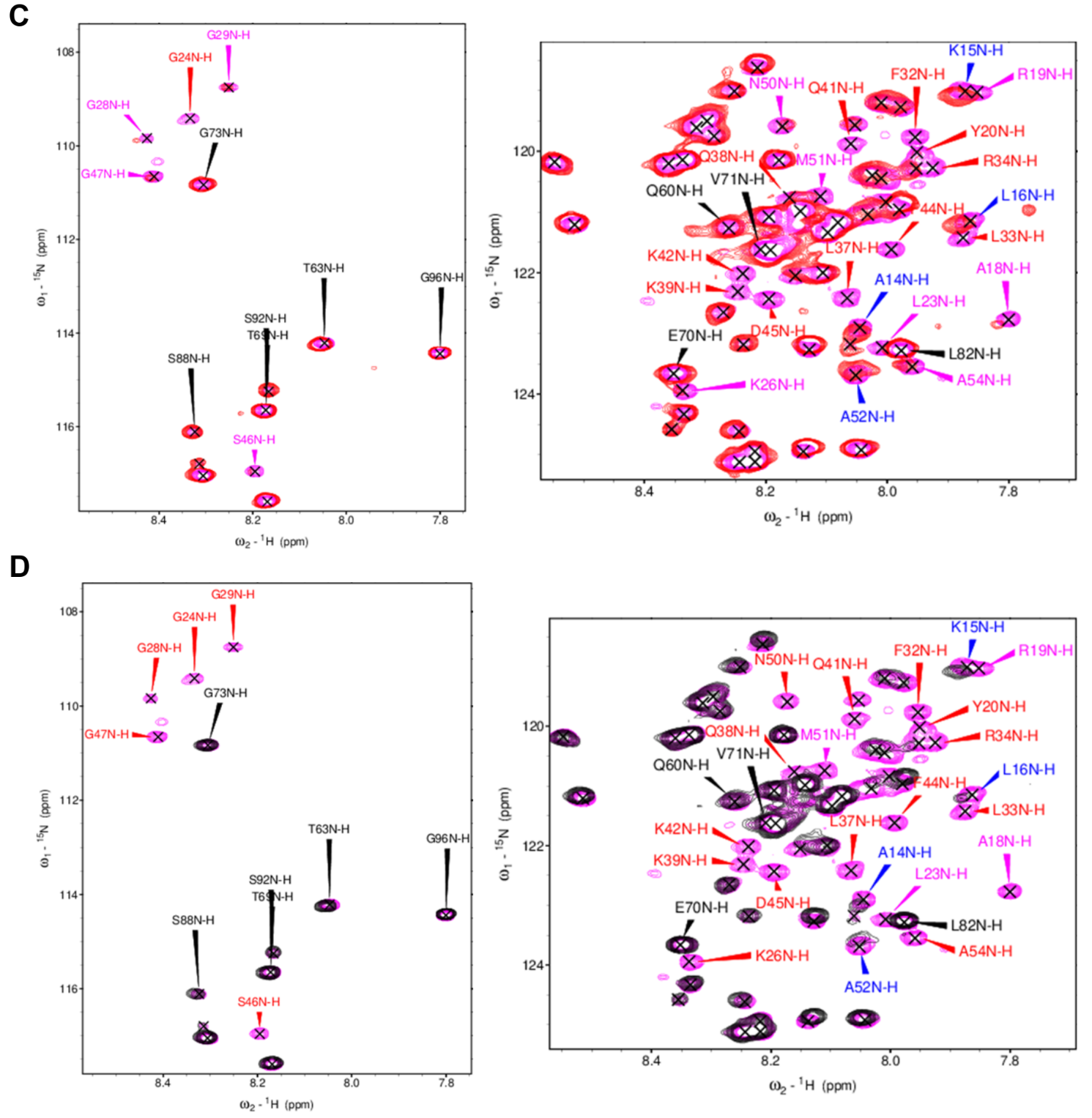
**

**Supplementary Figure 8.** Titration of PP2A A-subunit binds into the ARPP-16 related to the Figure 8A and 8B in this study. The overlaid of ^15^N-HSQC of ^15^N-labelled free ARPP-16 (magenta) and 1:0.5 (gold) (A), 1:1 (green) (B), 1:2 (red) (C), and 1:3 (black) ARPP-16 : PP2A A-subunit. The NH cross peaks that broadened with 1:2 PP2A A-subunit - ARPP-16 are labelled red, whereas the cross peaks that shifted the most and exhibit significant line broadening are labelled with magenta and the NH correlations with small CSPs are labelled blue.

**
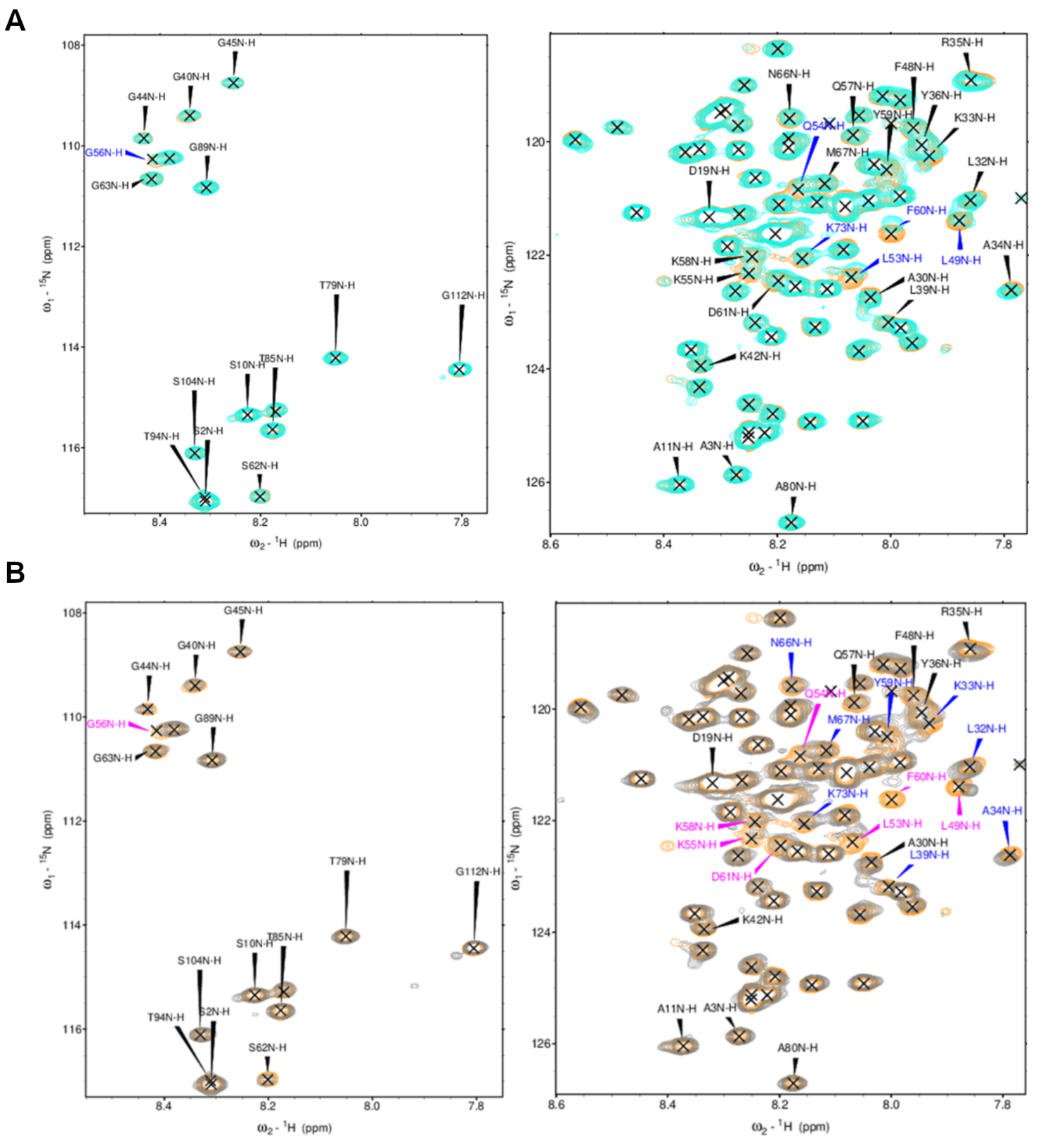
**

**
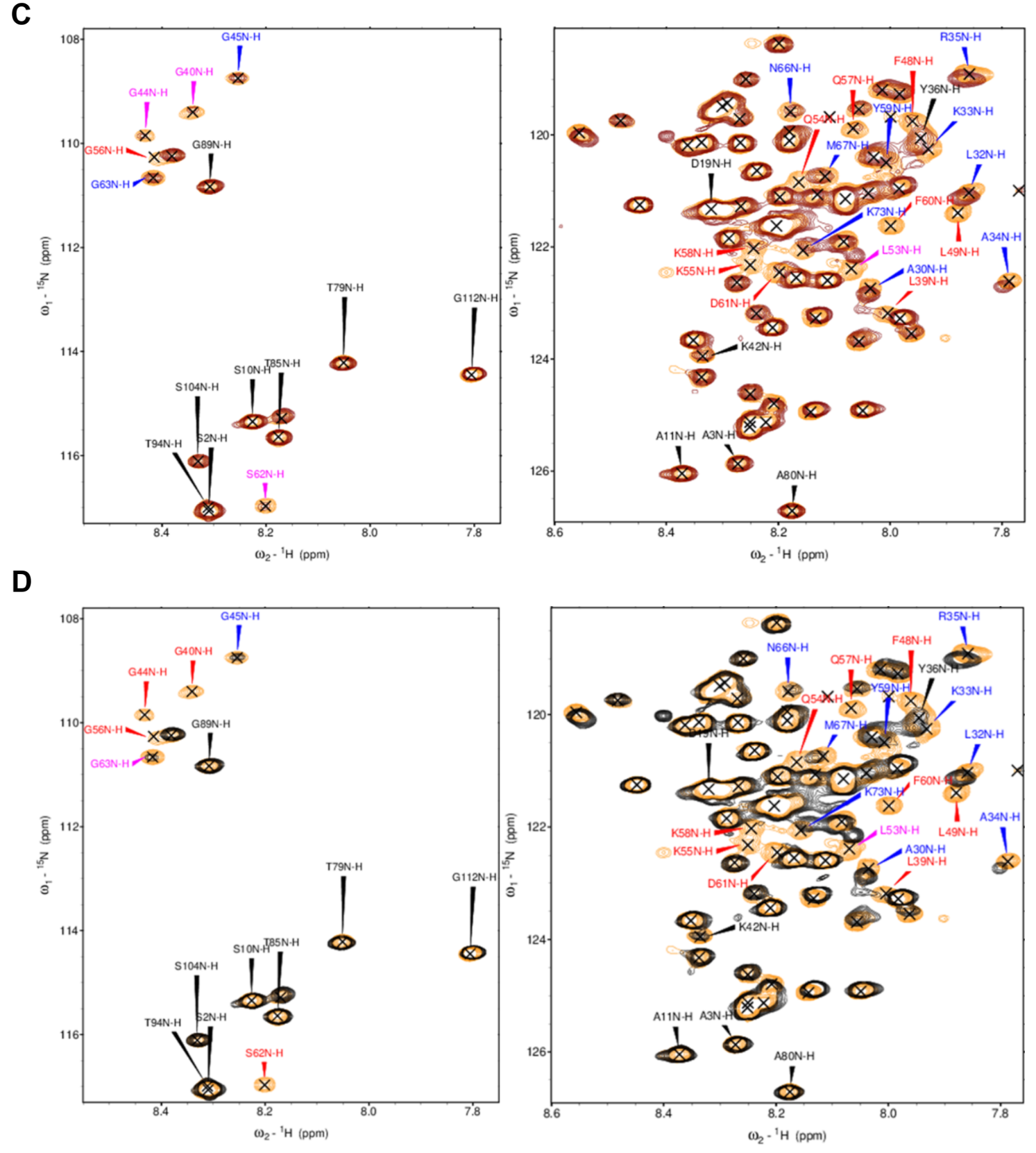
**

**Supplementary Figure 9.** Titration of PP2A A-subunit binds into the ARPP-19 related to the Figure 8C and 8D in this study. The overlaid of ^15^N-HSQC of ^15^N-labelled free ARPP-19 (orange) and 1:0.5 (cyan) (A), 1:1 (gray) (B), 1:2 (maroon) (C), and 1:3 (black) ARPP-19 : PP2A A-subunit. The NH cross peaks that broadened with 1:2 PP2A A-subunit - ARPP-19 are labelled red, whereas the cross peaks that shifted the most and exhibit significant line broadening are labelled with magenta and the NH correlations with small CSPs are labelled blue.

**References**

Bernadó, P., Mylonas, E., Petoukhov, M. V, Blackledge, M., and Svergun, D. I. (2007). Structural characterization of flexible proteins using small-angle X-ray scattering. *J. Am. Chem. Soc.* 129, 5656–64. doi:10.1021/ja069124n.

Konarev, P. V, Volkov, V. V, Sokolova, A. V, Koch, M. H. J., and Svergun, D. I. (2003). PRIMUS : a Windows PC-based system for small-angle scattering data analysis. *J. Appl. Crystallogr.* 36, 1277–1282. doi:10.1107/S0021889803012779.

Petoukhov, M. V, Konarev, P. V, Kikhney, A. G., and Svergun, D. I. (2007). ATSAS 2.1 - Towards automated and web-supported small-angle scattering data analysis. *J. Appl. Crystallogr.* 40, 223–228. doi:10.1107/S0021889807002853.

Tria, G., Mertens, H. D. T., Kachala, M., and Svergun, D. I. (2015). Advanced ensemble modelling of flexible macromolecules using X-ray solution scattering. *IUCrJ* 2, 207–217. doi:10.1107/S205225251500202X.
